# C9-ALS/FTD-linked proline–arginine dipeptide repeat protein associates with paraspeckle components and increases paraspeckle formation

**DOI:** 10.1038/s41419-019-1983-5

**Published:** 2019-10-03

**Authors:** Hiroaki Suzuki, Yoshio Shibagaki, Seisuke Hattori, Masaaki Matsuoka

**Affiliations:** 1 Department of Pharmacology, School of Medicine, Tokyo Medical University, 6-1-1 Shinjuku, Shinjuku-ku, Tokyo 160-8402 Japan; 2 Division of Biochemistry, School of Pharmaceutical Sciences, Kitasato University, 5-9-1 Shirokane, Minato-ku, Tokyo 108-8641 Japan

**Keywords:** Molecular biology, Neuroscience

## Abstract

A GGGGCC hexanucleotide repeat expansion in the *C9ORF72* gene has been identified as the most common genetic cause of amyotrophic lateral sclerosis and frontotemporal dementia. The repeat expansion undergoes unconventional translation to produce five dipeptide repeat proteins (DPRs). Although DPRs are thought to be neurotoxic, the molecular mechanism underlying the DPR-caused neurotoxicity has not been fully elucidated. The current study shows that poly-proline-arginine (poly-PR), the most toxic DPR in vitro, binds to and up-regulates nuclear paraspeckle assembly transcript 1 (NEAT1) that plays an essential role as a scaffold non-coding RNA during the paraspeckle formation. The CRISPR-assisted up-regulation of endogenous NEAT1 causes neurotoxicity. We also show that the poly-PR modulates the function of several paraspeckle-localizing heterogeneous nuclear ribonucleoproteins. Furthermore, dysregulated expression of TAR DNA-binding protein 43 (TDP-43) up-regulates NEAT1 expression and induces neurotoxicity. These results suggest that the increase in the paraspeckle formation may be involved in the poly-PR- and TDP-43-mediated neurotoxicity.

## Introduction

Amyotrophic lateral sclerosis (ALS) is a neurodegenerative disease that selectively affects motor neurons^1,2^. Frontotemporal dementia (FTD) is the second most common form of early-onset dementia, characterized by the degeneration of the frontal and temporal lobes^3,4^. Although it has been recognized that they share common pathological hallmarks, such as TAR DNA-binding protein 43 (TDP-43) aggregation^1,5^, the molecular mechanism underlying neurodegeneration in ALS/FTD has not been fully elucidated.

A GGGGCC hexanucleotide repeat expansion within the non-coding region of the gene encoding *C9ORF72* has been identified as the most common genetic cause of ALS and FTD (C9-ALS/FTD)^6,7^. Unconventional translation of the expanded repetitive sequence generates dipeptide repeat proteins (DPRs), consisting of poly-glycine-alanine (GA), poly-glycine-arginine (GR), poly-proline-arginine (PR), poly-proline-alanine (PA), and poly-glycine-proline (GP)^8^ and these DPRs can cause neurotoxicity^9^. We have previously shown that poly-PR is the most toxic DPR in vitro^10^. Currently, however, the mechanism underlying the DPR-linked neurotoxicity remains insufficiently characterized.

Paraspeckles are nuclear bodies that contain a long non-coding RNA (lncRNA), named nuclear paraspeckle assembly transcript 1 (NEAT1), as an essential scaffold RNA^11–13^ and more than 60 paraspeckle proteins^14–17^. There are two NEAT1 transcripts, NEAT1_1 (3.7 kb) and NEAT1_2 (22.7 kb), and the sequence of NEAT1_1 completely overlaps with the 5′ portion of NEAT1_2. Although it has been reported that paraspeckles participate in RNA metabolism^18–23^, the precise physiological function of paraspeckles remains unknown. It should be noted that some ALS/FTD-related proteins, the encoding genes of which have been identified as ALS/FTD-causative genes, have also been identified as paraspeckle proteins^14,16^. The dysregulation of paraspeckles has been implicated in some neurodegenerative diseases^24^. In particular, the increased NEAT1 expression and paraspeckle formation are observed in affected regions in patients with ALS/FTD including C9-ALS^25–28^. However, it remains unclear how the increased NEAT1 expression or paraspeckle formation is linked to neurodegeneration.

Our previous study has shown that poly-PR interacts with multiple paraspeckle proteins^10^, suggesting that poly-PR functionally affects paraspeckles. The present study shows that poly-PR up-regulates the expression of NEAT1, and the CRISPR-assisted up-regulation of endogenous NEAT1 expression causes neurotoxicity. These findings suggest that the up-regulation of NEAT1 may contribute to the poly-PR-caused neurotoxicity. In addition, this study demonstrates that poly-PR interacts with some paraspeckle-localizing heterogeneous nuclear ribonucleoproteins (hnRNPs) and dysregulates their function. We also show that poly-PR binds to TDP-43 and that the low-grade overexpression as well as the reduced expression of TDP-43 up-regulate NEAT1 expression. Collectively, these results suggest that the increase in the paraspeckle formation may be involved in the poly-PR- and the TDP-43-linked neurotoxicity.

## Materials and methods

### Antibodies

The following antibodies were used in this study: hnRNPF/H (sc-32310, RRID:AB_2248257), Splicing factor, proline- and glutamine-rich (SFPQ) (sc-374502, RRID:AB_10989589), non-POU domain-containing octamer-binding protein (NONO) (sc-166702, RRID:AB_2152178), hnRNPQ (sc-56703, RRID:AB_2200715), hnRNPA2/B1 (sc-374053, RRID:AB_10947257), and glutathione S-transferase (GST) (sc-138, RRID:AB_627677) from Santa Cruz Biotechnology (Dallas, TX); hnRNPM (A500–011A, RRID:AB_11125542) from Bethyl Laboratories (Montgomery, TX); horseradish peroxidase (HRP)-conjugated FLAG (A8592, RRID:AB_439702) from Sigma-Aldrich (St. Louis, MO); HA (11867423001, RRID:AB_390918) and HRP-conjugated HA (12013819001, RRID:AB_390917) from Roche Diagnostics (Basel, Swiss); monoclonal TDP-43 (89789, RRID:AB_2800143) and Glyceraldehyde 3-phosphate dehydrogenase (GAPDH) (2118, RRID:AB_561053) from Cell Signaling Technology (Danvers, MA); β-Tubulin (014–25041, RRID:AB_2650453) from FUJIFILM Wako Pure Chemical Corporation (Osaka, Japan); polyclonal TDP-43 (12892–1-AP, RRID:AB_2200505) and polyclonal Matrin3 (MATR3) (12202–2-AP, RRID:AB_2281752) from Proteintech Group (Rosemont, IL); HRP-conjugated goat anti-rabbit IgG (H + L) secondary antibody (170–6515, RRID:AB_11125142) and HRP-conjugated goat anti-mouse IgG (H + L) secondary antibody (170–6516, RRID:AB_11125547) from Bio-Rad Laboratories (Hercules, CA).

### Plasmids

The DPR cDNAs used in this study encode a 100-repeat of each DPR protein (DPR100)^10^. The FLAG-tagged PR100 (F-PR100) and EGFP-FLAG-tagged PR100 mammalian expression vectors were constructed as described previously^10^. GST-FLAG (GF)-tagged DPR100 bacteria expression vectors were constructed by inserting FLAG-tagged DPR100 cDNA^10^ into the pGEX-2T vector (GE Healthcare UK Ltd, Buckinghamshire, England). The cDNAs encoding human hnRNPF, hnRNPH1, hnRNPM, hnRNPQ, and family with sequence similarity 98 member A (FAM98A) were amplified from the human brain cDNA library (Takara, Shiga, Japan) and subcloned into the pEF4/His vector (Thermo Fisher Scientific, Waltham, MA), in which the HA tag-encoding sequence was inserted before the Xpress tag-encoding sequence to express HA-tagged hnRNPF, hnRNPH1, hnRNPM, hnRNPQ, and FAM98A, respectively. The human RNA-binding motif protein 14 (RBM14)-encoding plasmid, kindly provided by Dr. Archa H. Fox (The University of Western Australia)^29^, and the human MATR3 cDNA, a gift from Dr. Yossi Shiloh (Addgene plasmid # 32880, RRID:Addgene_32880)^30^, were subcloned into the pEF4/His vector to express HA-tagged RBM14 and MATR3, respectively. pAC152-dual-dCas9VP64-sgExpression and U6-sgRNA(MS2)_EF1a-MS2-P65-HSF1 were gifted from Dr. Rudolf Jaenisch (Addgene plasmid # 48238, RRID:Addgene_48238)^31^ and Dr. Ervin Welker (Addgene plasmid # 92120, RRID:Addgene_92120)^32^, respectively. For construction of single guide (sg) RNA that recruits the synergistic activation mediator complex on the *NEAT1* promoter, the sense primer (5′-CACCGAGGGAGTGGCGGTTGACGG-3′) and the antisense primer (5′-AAACCCGTCAACCGCCACTCCCTC-3′) were phosphorylated by T4 polynucleotide kinase and in vitro annealed and were then subcloned into the *Bsa*I site of the U6-sgRNA(MS2)_EF1a-MS2-P65-HSF1 vector. The target of sgRNA is located at −172–−153 in the *NEAT1* promoter region (the transcription start site is defined as +1). pCMV-HA-TDP-43 was provided by Dr. Randal S. Tibbetts (University of Wisconsin). The TDP-43 cDNA was subcloned into the pEF1/Myc-His vector (Thermo Fisher Scientific) with a native stop codon or the pEF4/His vector in order to construct non-tagged (pEF1-TDP-43) or the HA-tagged TDP-43 expression vector, respectively. HA-tagged TDP-43-(1–273) (Δlow-complexity domain, LCD) and HA-tagged RBM14-(1–349) (ΔLCD) were generated using the KOD-Plus-Mutagenesis kit (Toyobo, Osaka, Japan). The expression vectors of mouse NEAT1_1 (3.2 kb), NEAT1_2 (20.7 kb), and their control vector (pCMV-GFP) were kindly provided by Dr. Nobuyoshi Akimitsu (The University of Tokyo)^21^.

### Cell culture and transfection

NSC-34 motor neuronal cells (RRID:CVCL_D356), established from mouse neuroblastoma cells and mouse embryo spinal cord cells, were a kind gift from Dr. Neil Cashman (University of Toronto) and were authenticated by microscopic cell morphology and growth rate analysis. HEK293 cells (RRID:CVCL_0045) were acquired from our laboratory depository and were authenticated by microscopic cell morphology and the capability of adenovirus generation. The absence of mycoplasma contamination was validated using a MycoAlert™ Mycoplasma Detection Kit (Takara). HeLa cells were purchased from ATCC (Manassas, VA) (CCL-2, RRID:CVCL_0030). Cells were grown in Dulbecco’s modified Eagle’s medium (DMEM) (FUJIFILM Wako Pure Chemical Corporation), supplemented with 10% of fetal bovine serum (FBS) (GE Healthcare UK Ltd) and antibiotics (Thermo Fisher Scientific). Transfection was performed using Lipofectamine (Thermo Fisher Scientific) and PLUS reagent (Thermo Fisher Scientific) or Lipofectamine 2000 (Thermo Fisher Scientific) under the manufacturer’s protocol.

### Adenovirus vector-mediated expression

The adenovirus expression vector systems were purchased from Takara. Adenovirus vectors encoding LacZ, Cre recombinase, FLAG-tagged GA100, GR100, PR100, PA100, TDP-43-wt, -ΔNLS (Δ82–98), -2FL (F147L/F149L), -6GGGG (substitution of 6R7V8T9E with 6G7G8G9G), and -(90–414) were constructed as described previously^10,33,34^. The cDNAs encoding NEAT1_1, hnRNPF, hnRNPH1, hnRNPM, hnRNPQ, and MATR3 were inserted into the *Swa*I site of a cosmid adenoviral vector, pAxCALNLw. In this vector, a stuffer DNA fragment, sandwiched between two loxP sequences, is located just at the upstream of cDNA and interferes with gene expression. If an adenovirus vector expressing Cre recombinase is co-introduced into the cells, the stuffer is removed, and the gene is expressed. All viruses were grown in HEK293 cells and were purified using CsCl gradient ultracentrifugation. The adenovirus titers were determined by measuring the absorbance (Abs) of adenovirus DNA at 260 nm wavelength. The biologically active viruses are thought to be less than those indicated as multiplicity of infection (MOI). NSC-34 cells, seeded at 1 × 10^5^ cells/well on 6-well plates or 5 × 10^4^ cells/well on 12-well plates, or HeLa cells, seeded at 5 × 10^4^ cells/well on 6-well plates or 2.5 × 10^4^ cells/well on 12-well plates, were incubated with media containing adenovirus vector at the indicated MOI at 37 °C. Unless otherwise indicated, cells were infected with adenovirus encoding Cre-recombinase at an MOI of 40.

### Primary neurons

Primary cultured cerebral cortical neurons (PCNs), obtained from ICR mice on embryonic day 14, were prepared, as described previously^35,36^. Briefly, cerebral cortices were dissected and digested with 800–900 U/mL papain (Sigma-Aldrich) in the Hanks’ Balanced Salt Solution (Thermo Fisher Scientific) containing 0.2 mg/mL l-cysteine (FUJIFILM Wako Pure Chemical Corporation), 0.2 mg/mL bovine serum albumin (Sigma-Aldrich), and 5 mg/mL glucose (FUJIFILM Wako Pure Chemical Corporation) at 37 °C for 10 min. After the digestion, the cells were dispersed by gentle pipetting in DMEM supplemented with 10% FBS and were filtered through 40 μm Nylon cell strainer (BD Falcon, Corning, NY). The filtered cells were collected by centrifugation and re-suspended in Neuron medium (Sumitomo Bakelite, Tokyo, Japan). The cells were then counted in a hemocytometer and their viability was assayed by trypan blue exclusion test (Sigma-Aldrich). Viable cells were seeded on poly-l-lysine-coated 6-well plates (Sumitomo Bakelite) at 1 × 10^6^ cells/well in Neuron medium and were maintained at 37 °C, 5% CO_2_, and in saturated humidity. By this procedure, neurons represented more than 98% of the cells present in cultures, as described in the original reference and in our previous study^36,37^. On day in vitro 3 (DIV3), PCNs were infected with adenoviruses at the indicated MOI in Neuron medium at 37 °C. All animal experiments were approved by the Institutional Animal Care and Use Committee of Tokyo Medical University (No. H300056) and were conducted in accordance with the Society’s Policies on the Use of Animals in Neuroscience Research.

### Cell viability assay

Cell viability was measured by WST-8 cell viability assays. The WST-8 assay, performed using Cell Counting Kit-8 (Dojindo, Osaka, Japan), is based on the ability of cells to convert a water-soluble WST-8 into a water-soluble WST-8 formazan. Cells were treated with WST-8 reagent at 37 °C, and Abs was measured at 450 nm by a multilabel reader 2030 ARVO™ X5 (Perkin Elmer, Waltham, MA). The value of Abs at 450 nm is increased in proportion to cell numbers, so that the WST-8 assay is capable of estimating the numbers of living cells (Fig. S1).

### Western blotting and dot blotting analysis

Cells were lysed in a cell lysis buffer (10 mM Tris–HCl [pH 7.4], 1% Triton X-100, 1 mM EDTA, and protease inhibitors) by a freeze-thaw cycle or in a 4% SDS-containing sample buffer by sonication. Protein concentration was measured by Pierce BCA Protein Assay Kit (Thermo Fisher Scientific). The samples in the SDS-containing sample buffer were boiled at 95 °C for 5 min, fractionated by SDS–PAGE, and blotted onto polyvinylidene fluoride (PVDF) membranes (Pall Corporation, Port Washington, NY). For dot blotting analysis, 1 μL of cell lysates in a 4% SDS-containing sample buffer was spotted onto PVDF membranes. After blocking with 5% skim milk (Becton, Dickinson and Company, Sparks, MD) in Tris-buffered saline with 0.1% Tween 20 (TBST), immunoblotting was performed with indicated antibodies. Immunoreactive bands were detected with ECL Western Blotting Detection Reagents (GE Healthcare UK Ltd). GAPDH or β-Tubulin was used as an internal control. Intensities of immunodetected signals were densitometrically measured with an ImageJ software (Ver. 1.51). As shown in our previous study^10^, dot blotting analysis is more suitable than western blotting analysis for the detection of DPRs expression levels; thus, we measured the total levels of DPRs by dot blotting analysis.

### Pull-down assay

Recombinant proteins were produced using *Escherichia coli* BL21(DE3)pLysS competent cells (Promega, Madison, WI) as the host and the pGEX vector system (GE Healthcare UK Ltd). NSC-34 cells or NSC-34 cells, transiently transfected with indicated vectors for 48 h, were harvested and lysed in a pull-down buffer (150 mM NaCl, 20 mM HEPES [pH 7.4], 1 mM EDTA, 1 mM DTT, 0.1% Triton X-100, and protease inhibitors) by sonication. After centrifugation at 12 000 g for 15 min, the cell lysates were pre-cleared with recombinant GST-bound glutathione beads for 1.5 h and were then incubated with recombinant GST or GST-FLAG-DPR100-bound glutathione beads at 4 °C by rotation. For RNase treatment, the pre-cleared supernatants and recombinant GST or GST-FLAG-PR100-bound glutathione beads were incubated with or without 20 μg/mL RNase A (FUJIFILM Wako Pure Chemical Corporation) at 25 °C for 2.5 h in a pull-down buffer before mixing the cell lysates and recombinant protein-bound beads. After washing four times using the pull-down buffer, the precipitates were fractionated by SDS–PAGE, followed by immunoblotting analysis. The amounts of recombinant GST-FLAG-DPR100 proteins were estimated by staining with Coomassie Brilliant Blue G (Sigma-Aldrich) (Fig. S2b).

### Immunocytochemistry

NSC-34 cells were fixed with 4% paraformaldehyde-phosphate-buffered saline (PBS) and immunostained with the primary antibodies (hnRNPF/H, TDP-43, or HA antibodies) and the respective secondary antibodies (Cy3-labeled goat anti-mouse IgG [115–165–166, RRID:AB_2338692, Jackson ImmunoResearch Inc., West Grove, PA], Cy3-labeled goat anti-rabbit IgG [111–165–144, RRID:AB_2338006, Jackson ImmunoResearch Inc.], or Alexa Fluor 568-labeled goat anti-rat IgG [A11077, RRID:AB_2534121, Thermo Fisher Scientific]). The cells were then mounted using Hard Set Mounting Medium with 4′,6-diamidino-2-phenylindole (DAPI) (Vector Laboratories, Inc., Burlingame, CA). The cells were observed and analyzed using a confocal microscope LSM710 with ZEN 2010 software (Carl Zeiss, Oberkochen, Germany).

### RNA immunoprecipitation assay

RNA immunoprecipitation (RIP) assay was performed with Magna RIP RNA-Binding Protein Immunoprecipitation Kit (Merck Millipore, Burlington, MA) using the manufacturer’s protocol. Briefly, NSC-34 cells, transfected with the empty vector or the FLAG-PR100-encoding vector for 48 h, were lysed in a complete RIP lysis buffer. After centrifugation at 12 000 g for 10 min, the cell lysates were incubated with normal mouse IgG1 (sc-3877, RRID:AB_737222, Santa Cruz Biotechnology) or anti-FLAG antibody (F1804, RRID:AB_262044, Sigma-Aldrich)-bound Magnetic beads overnight at 4 °C by rotation. After washing six times with a cold RIP wash buffer, RNA was extracted from precipitates. First-strand cDNAs were synthesized from purified RNA using QuantiTect Rev. Transcription Kit (QIAGEN, Hilden, Germany). PCR amplification with KOD-Plus-Ver.2 (TOYOBO) was performed using a Mastercycler X50s (Eppendorf, Hamburg, Germany) under initial denaturation at 94 °C for 2 min, followed by 22–30 cycles of denaturation at 98 °C for 10 s, annealing at 55 °C for 30 s, and extension at 68 °C for 30 s. The sequences of forward and reverse primers are presented as follows: NEAT1_1 & NEAT1_2, sense: 5′-GGGGCCACATTAATCACAAC-3′, antisense: 5′-CTCAGAGTGAGGGGCAAGAG-3′; NEAT1_2, sense: 5′-ATGAGGCACAAATGGAGGTC-3′, antisense: 5′-TCAGCCCCAAGATCGATAAC-3′; Malat1, sense: 5′-GCGGGTGTTGTAGGTTTTTC-3′, antisense: 5′-ACAAAACTGGGAGGTTGTGC-3′; Xist, sense: 5′-GCCTCTGATTTAGCCAGCAC-3′, antisense: 5′-ATGCAACCCCAGCAATAGTC-3′. PCR amplicons were validated by sequence analysis. Five percent of the final IP samples were used as input. In parallel with RIP assay, a part of input and IP samples was subjected to dot blotting analysis.

### RNA fluorescence in situ hybridization and flow cytometry

RNA fluorescence in situ hybridization (FISH) assay was conducted using Stellaris RNA (LGC Biosearch Technologie, Petaluma, CA) under the manufacturer’s protocol. Briefly, NSC-34 cells, transfected with indicated vectors using Lipofectamine 2000 (Thermo Fisher Scientific) under the manufacturer’s protocol, were fixed with 3.7% formaldehyde-PBS at 48 h after the transfection. The fixed cells were then immersed in 70% ethanol for permeabilization at 4 °C overnight. After washing with wash buffer A (LGC Biosearch Technologie) at 25 °C for 5 min, cells were incubated with a hybridization buffer (LGC Biosearch Technologie) containing 10% deionized formamide and Quasar 570-labeled 125–1000 nM Stellaris probe NEAT1 5’ Seg. (LGC Biosearch Technologie) that detects both NEAT1_1 and NEAT1_2 at 37 °C for 4 h. Following incubation, the cells were washed with the wash buffer A at 37 °C for 30 min and were then mounted using Hard Set Mounting Medium with DAPI (Vector Laboratories). The cells were observed and analyzed using a confocal microscope LSM710 with ZEN 2010 software (Carl Zeiss).

Separately, the suspension cells (1–2 × 10^6^ cells/200 μL for each sample) were stained for flow cytometry analysis. The fluorescence was measured by UNITECH Co., Ltd. (Kashiwa, Chiba, Japan) with BD FACSAria Special Order Research Products (BD Biosciences, Franklin Lakes, NJ) and was analyzed with BD FACSDiva ver. 8.0.1 (BD Biosciences). Gating strategies for the flow cytometry are shown in Fig. S4d.

### Quantitative real-time PCR analysis

Total RNA was extracted from NSC-34 cells or HeLa cells infected with indicated adenovirus vectors or transfected with indicated siRNAs or expression vectors using RNeasy Plus Mini kit (QIAGEN). Genomic DNA was minimized with gDNA eliminator spin columns and DNase treatment using RNase-Free DNase Set (QIAGEN). Reverse transcription and PCR reactions were performed on an Applied Biosystems StepOnePlus™ Real-Time PCR System (Thermo Fisher Scientific) using the Taqman RNA-to-Ct 1-Step Kit (Thermo Fisher Scientific). The pairs of primers and the Taqman probes for target RNAs were designed based on mouse and human RNA sequences using Taqman Gene Expression Assays (Thermo Fisher Scientific). Assay IDs of Taqman probes for mouse Neat1, Neat1_2, Hnrnpm, Nfkb1, Nr4a1, and Gapdh are Mm03942186_s1, AJT96WW (Custom Plus TaqMan® RNA Assays), Mm00513070_m1, Mm00476361_m1, Mm01300401_m1, and Mm99999915_g1, respectively. Mm03942186_s1 was used to detect both Neat1_1 and Neat1_2, whereas AJT96WW was used to specifically detect Neat1_2. Assay IDs of Taqman probes for human NEAT1, CCL5, CXCL8, SH3PXD2A, TSHZ2, and GAPDH are Hs03453534_s1, Hs00982282_m1, Hs00174103_m1, Hs01046307_m1, Hs00542836_m1, and Hs02758991_g1, respectively. Hs03453534_s1 detects both NEAT1_1 and NEAT1_2. Data analysis was performed using StepOne Software Ver. 2.0.2 (Thermo Fisher Scientific). Relative expression was analyzed by the relative standard curve method. Data were normalized to the mRNA expression of GAPDH.

### Luciferase assay

A *NEAT1*-promoter luciferase vector, kindly provided by Dr. Nobuyoshi Akimitsu (The University of Tokyo)^21^, is a reporter plasmid containing the luciferase gene under the control of the human *NEAT1* promoter (−951– + 110; the transcription start site is defined as +1). For the construction of the *NEAT1*-promoterless luciferase control vector, the *NEAT1* promoter sequence was deleted by digestion with *Xho*I and *Hind*III. NSC-34 cells, seeded on 24-well plates at 4 × 10^4^ cells/well, were transfected with 0.0625 μg/well of the luciferase vector or the *NEAT1*-promoterless luciferase vector together with 0.25 μg/well of pEF1-Myc/His-vec, FLAG-PR100-, hnRNPM-, or the TDP-43-encoding vector. At 48 h after the transfection, luciferase assays were performed with the Dual-Luciferase Reporter Assay (Promega) using Lumat LB 9507 luminometer (Berthold Technologies, Bad Wildbad, Germany). The pGL4-TK *Renilla* luciferase vector (Promega) (0.0625 μg/well) was co-transfected to monitor transfection efficiency. Calculated luciferase activities were normalized by transfection efficiency.

### siRNA- and antisense oligonucleotide-mediated silencing

siRNAs against mouse hnRNPF, hnRNPH1, and TDP-43 and non-targeting control siRNA were purchased from RNAi Co., Ltd. (Tokyo, Japan). The siRNA sequences for mouse hnRNPF, hnRNPH1, and TDP-43 are 5′-CCGCUUUUCUCAAUUAACAUU-3′, 5′-CUCAAUGUUGAGCAUAGCAGU-3′, and 5′-GGAAUCAGCGUGCAUAUAUCC-3′, respectively. The sequence of antisense oligonucleotides (ASOs) against NEAT1 and a control ASO were derived from a previous report^11^. ASOs were synthesized by Integrated DNA Technologies, Inc. (Skokie, IL). The ASO sequences for NEAT1 and control are 5′-mC*mC*mC*mA*mG*T*C*C*A*C*C*C*G*T*C*mU*mC*mC*mA*mU-3′ and 5′-mU*mC*mA*mC*mC*T*T*C*A*C*C*C*T*C*T*mC*mC*mA*mC*mU-3′, respectively. The asterisks represent the phosphorothioate-modified backbone and mN designates the 2′-O-methoxyribonucleotides. Another ASO LNA GapmeR, purchased from QIAGEN, were also used for NEAT1 knock-down (control: LG00000002-DDA, Lot# 11100372, NEAT1: LG00202797-DDA, Lot# 37209579). NSC-34 cells were transfected using Lipofectamine 2000 (Thermo Fisher Scientific) according to the manufacturer’s reverse transfection protocol. Briefly, 6 × 10^4^ cells/well on 6-well plates or 3 × 10^4^ cells/well on 12-well plates were combined with 5 nM siRNA or 30 nM ASO and Lipofectamine 2000 reagent complexes.

### Statistics analysis

All experiments in the figure were basically conducted more than 2 times. The image of immunocytochemical analysis and RNA FISH analysis is representative of more than 4 images. All values in the figures are shown as means ± SD. All experiments that were statistically analyzed were performed with three biological replicates. No statistical method was used to predetermine the sample size. Sample sizes in this study were estimated based on previous experience that showed significance and standard in the field. Statistical analysis was performed with a one-way analysis of variance (ANOVA), followed by post-hoc test (Dunnett’s or Tukey’s multiple comparisons test) or two-tailed Student’s *t*-test. A confidence interval was set at 95% and a *p* value of <0.05 was used as the cutoff for statistical significance. All statistics analyses were conducted using Prism 7 software (Ver. 7.0d) (*p < 0.05; **p < 0.01; ***p < 0.001; ****p < 0.0001; ns, not significant).

## Results

### Poly-PR interacts with paraspeckle components

**Fig. 1:**
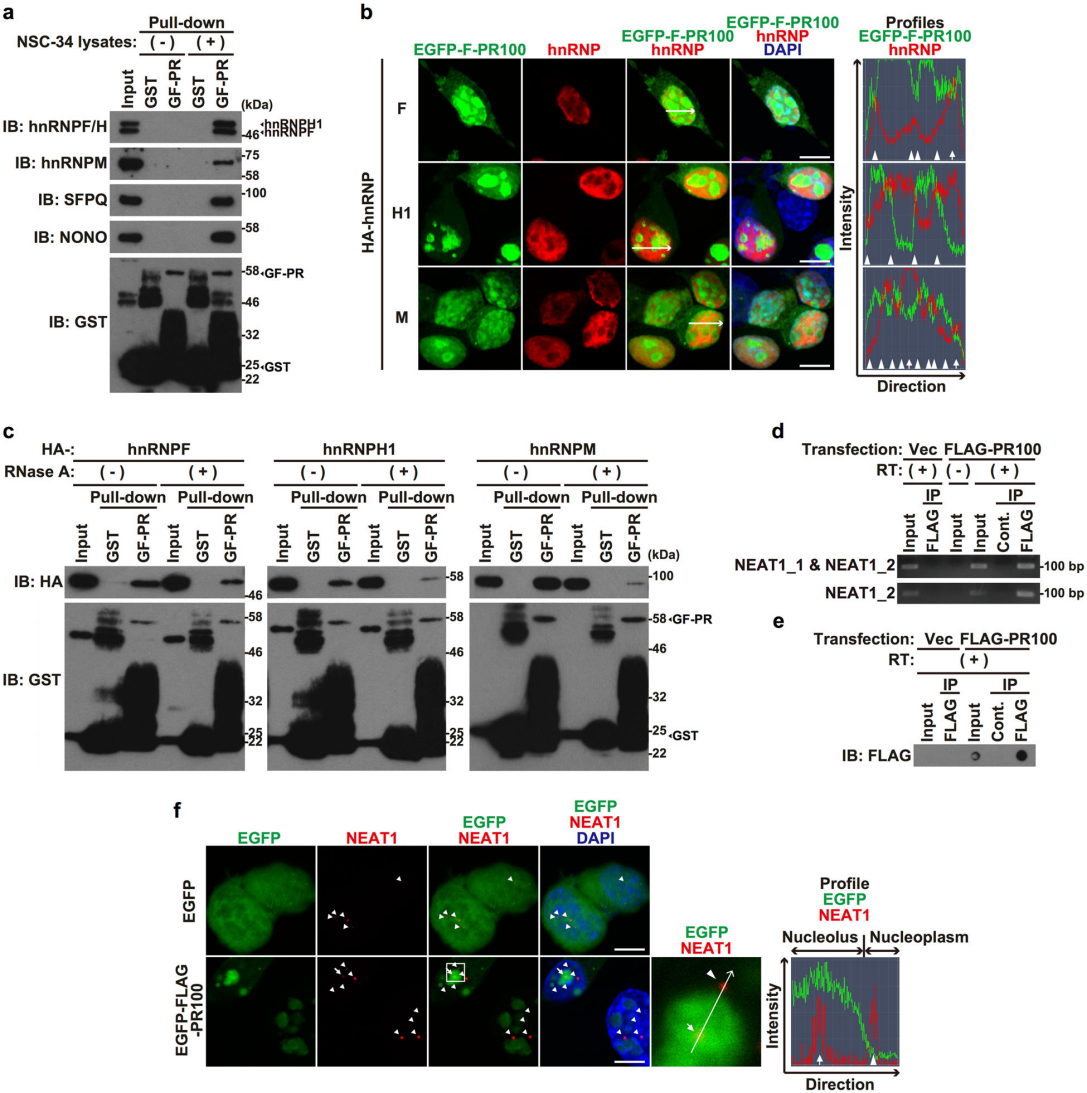
Poly-proline-arginine (Poly-PR) associates with paraspeckle components. **a** Glutathione S-transferase (GST) or GST-FLAG-PR100 (GF-PR)-bound glutathione beads were mixed with ( + ) or without (−) NSC-34 cell lysates. After the rotation at 4 °C for 5 h, the glutathione beads were washed and were subjected to 5–20% gradient gel SDS–PAGE followed by immunoblotting (IB) using indicated antibodies. In western blotting using anti-GST antibody, the large smear within the molecular weights ranging 25–46 kDa in the GF-PR lane is thought to consist of C-terminal truncated GST-FLAG-PR100 proteins. A band located around 50 kDa in the GST lane is thought to represent dimerized and/or aggregated GST-derived proteins. **b** NSC-34 cells overexpressing EGFP-FLAG-PR100 (EGFP-F-PR100) (green) together with HA-tagged hnRNPF, hnRNPH1, or hnRNPM were fixed and were immunostained with HA (red). Nuclei were stained with 4′,6-diamidino-2-phenylindole (DAPI, blue). Scale bar = 10 μm. The rightmost panel shows the profile image of fluorescence intensities on the lines of EGFP-FLAG-PR100 and hnRNPs-merged image. Arrows and arrowheads indicate the localization of nucleoplasm-localizing EGFP-FLAG-PR100 and the border of nucleolus-localizing EGFP-FLAG-PR100, respectively. **c** Lysates of NSC-34 cells overexpressing HA-tagged hnRNPF, hnRNPH1, or hnRNPM and GST or GST-FLAG-PR100 (GF-PR)-bound glutathione beads were incubated with ( + ) or without (−) 20 μg/mL RNase A. After the incubation, cell lysates were mixed with GST or GST-FLAG-PR100-bound glutathione beads. The glutathione beads were washed and were subjected to immunoblotting (IB) using indicated antibodies. **d**, **e** NSC-34 cells were transfected with the empty vector or the FLAG-PR100-encoding vector. At 48 h after the transfection, the cell lysates were immunoprecipitated (IP) with normal mouse IgG1 (Cont.) or the FLAG antibody. Precipitates were then used for RNA immunoprecipitation (RIP) assay (**d**) and dot blotting analysis with the FLAG antibody (**e**). Reverse transcription (RT) (−) was used as negative control to monitor the PCR amplification from genomic DNA. **f** NSC-34 cells overexpressing EGFP or EGFP-FLAG-PR100 (green) were fixed and stained with the 500 nM Quasar 570-labeled NEAT1 Stellaris probe (red). Nuclei were stained with DAPI (blue). Scale bar = 10 μm. Arrowheads and arrow correspond to paraspeckles. The rightmost panel shows the profile image of fluorescence intensities on the lines of EGFP-FLAG-PR100 and NEAT1-merged image in magnified image. Arrowhead and arrow indicate the co-localization of NEAT1 with nucleoplasm-localizing EGFP-FLAG-PR100 and the nucleolus-localizing EGFP-FLAG-PR100, respectively

We have previously identified some paraspeckle proteins as putative poly-PR-binding proteins (Table S1)^10^. In the current study, using GST pull-down assay with GST-FLAG-tagged PR100 as a bait, we confirmed the interaction between poly-PR and endogenous or exogenous paraspeckle proteins, hnRNPF, hnRNPH1, hnRNPM, SFPQ, NONO, FAM98A, RBM14, and MATR3^14,16,38^ (Fig. 1a, c, and S2a). These paraspeckle proteins also interacted with poly-GR, but not with other DPRs, poly-GA or poly-PA (Fig. S2b,c). An RNA-binding protein hnRNPA2, which is not classified as a paraspeckle protein, hardly bound to poly-PR (Fig. S2c). Immunocytochemical analysis showed that exogenous hnRNPF, hnRNPH1, and hnRNPM co-localized with nucleoplasm-localizing EGFP-FLAG-PR100 (Fig. 1b, right panel, arrows in profile images, and Fig. S3a–c) and formed ring-like structure around poly-PR aggregates that likely correspond to the nucleolus^10^ (Fig. 1b, right panel, arrowheads in profile images, and Fig. S3a–c). Endogenous hnRNPF and hnRNPH1 also partially co-localized with EGFP-FLAG-PR100 in a similar pattern (Fig. S2d). The digestion of RNA with RNase A markedly reduced the interaction between poly-PR and hnRNPs (Fig. 1c), indicating that these interactions are partially dependent on the presence of RNAs.

We next examined whether poly-PR also bound to NEAT1, an essential scaffold RNA required for the paraspeckle formation^11–13^. RIP assay showed that poly-PR bound to NEAT1 (Fig. 1d, e). We also recognized the interaction between poly-PR and another lncRNAs, Malat1 and Xist (Fig. S2e). All these results suggest that poly-PR may have a propensity to bind to RNAs, as reported previously^39^. We also examined whether poly-PR co-localized with NEAT1. RNA FISH assay showed that the majority of NEAT1 fluorescent signals overlapped with nucleoplasm-localizing poly-PR (Fig. 1f, profile image arrowhead). Some NEAT1 signals also co-localized with poly-PR possibly in the nucleolus^10^ (Fig. 1f, profile image arrow). Consistently, two exogenous NEAT1 variants were shown to be co-localized with poly-PR (Fig. S2f).

### Poly-PR up-regulates NEAT1 expression

**Fig. 2:**
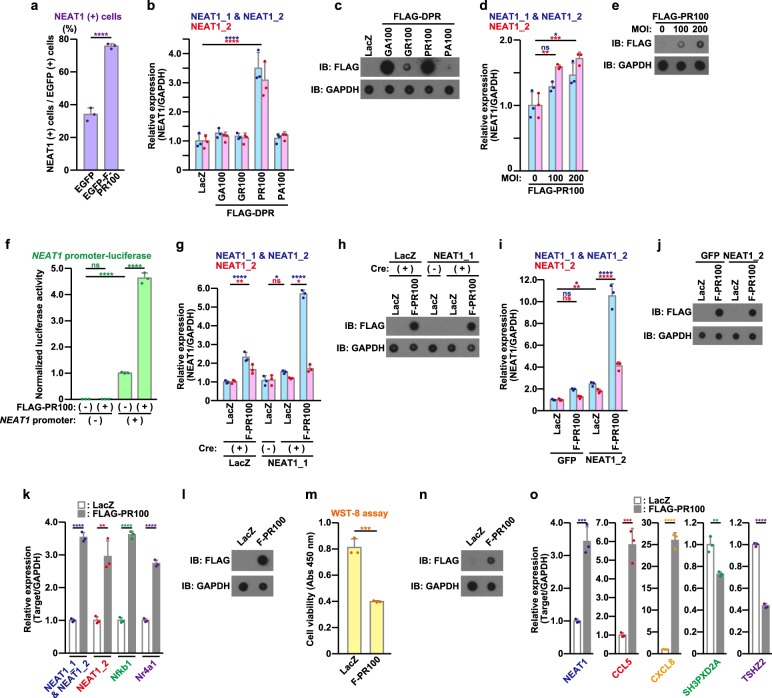
Poly-PR induces nuclear paraspeckle assembly transcript 1 (NEAT1) expression. **a** The number of EGFP- or EGFP-FLAG-PR100 (EGFP-F-PR100)-expressing paraspeckle-positive NSC34 cells in RNA FISH assay was counted (100 cells/count). The data are presented as means ± SD (*N* = 3). Statistical analysis was performed by the unpaired *t*-test. **b, c** NSC-34 cells were infected with indicated adenovirus vectors at a multiplicity of infection (MOI) of 800. At 48 h after the infection, the quantitative real-time PCR analysis of NEAT1 was performed (**b**). The cell lysates were subjected to dot blotting analysis using indicated antibodies (**c**). The data are presented as means ± SD (*N* = 3). Statistical analysis was performed by one-way analysis of variance (ANOVA) followed by the Dunnett’s multi comparisons test. **d**, **e** Primary cultured cerebral cortical neurons (PCNs) were infected with adenovirus encoding FLAG-PR100 at MOIs of 0–200. To keep the constant total MOIs of adenoviruses, appropriate MOIs of LacZ-encoding adenovirus were added for each infection. At 120 h after the infection, the quantitative real-time PCR analysis of NEAT1 was performed (**d**). The cell lysates were subjected to dot blotting analysis using indicated antibodies (**e**). The data are presented as means ± SD (*N* = 3). Statistical analysis was performed by one-way ANOVA followed by the Dunnett’s multi comparisons test. **f** NSC-34 cells were transfected with the *NEAT1*-promoter (+) or *NEAT1*-promoterless (−) luciferase vector together with the pEF1-Myc/His-vec (−) or the pEF1-FLAG-PR100 (+). At 48 h after the transfection, the luciferase activity was measured. The data are presented as means ± SD (*N* = 3). Statistical analysis was performed by one-way ANOVA followed by the Tukey’s multi comparisons test. **g**, **h** NSC-34 cells were infected with adenovirus encoding LacZ or mouse NEAT1_1 at an MOI of 1. Cells were also co-infected with adenovirus encoding LacZ or FLAG-PR100 at an MOI of 200 together with adenovirus encoding LacZ (−) or Cre-recombinase (+) at an MOI of 40. At 48 h after the infection, the quantitative real-time PCR analysis of NEAT1 was performed (**g**). The cell lysates were subjected to dot blotting analysis using indicated antibodies (**h**). The data are presented as means ± SD (*N* = 3). Statistical analysis was performed by one-way ANOVA followed by the Dunnett’s multi comparisons test. **i**, **j** NSC-34 cells were transfected with 0.2 μg/well of the pCMV-GFP or -mouse NEAT1_2 on 6-well plate. After the transfection, NSC-34 cells were infected with adenovirus encoding FLAG-PR100 at an MOI of 200. Total adenoviruses infected were adjusted at an MOI of 400 with adenovirus encoding LacZ. At 48 h after the infection, the quantitative real-time PCR analysis of NEAT1 was performed (**i**). The cell lysates were subjected to dot blotting analysis using indicated antibodies (**j**). The data are presented as means ± SD (*N* = 3). Statistical analysis was performed by one-way ANOVA followed by the Dunnett’s multi comparisons test. **k**, **l** NSC-34 cells were infected with adenovirus encoding LacZ or FLAG-PR100 at an MOI of 800. At 48 h after the infection, the quantitative real-time PCR analysis of NEAT1, Nfkb1, and Nr4a1 was performed (**k**). The cell lysates were subjected to dot blotting analysis using indicated antibodies (**l**). The data are presented as means ± SD (*N* = 3). Statistical analysis was performed by the unpaired *t*-test. **m–o** HeLa cells were infected with adenovirus encoding LacZ or FLAG-PR100 at an MOI of 800. At 48 h after the infection, the cell viability was detected by WST-8 assay (**m**). The cell lysates were subjected to dot blotting analysis using indicated antibodies (**n**). The quantitative real-time PCR analysis of NEAT1, CCL5, CXCL8, SH3PXD2A, and TSHZ2 was performed (**o**). The data are presented as means ± SD (*N* = 3). Statistical analysis was performed by the unpaired *t*-test

We next assessed whether poly-PR affected the NEAT1 expression. The percentage of NEAT1-positive cells by RNA FISH and the intensity of NEAT1 fluorescence signals by flow cytometry analysis were higher in the cells expressing EGFP-FLAG-PR100 than in those expressing EGFP (Fig. 2a, S4a, b, population P3–P7). These results together suggested that poly-PR up-regulated the NEAT1 expression and increased the number of paraspeckles. Quantitative real-time PCR analysis confirmed that poly-PR increased the NEAT1 expression in NSC-34 cells (Fig. 2b, c) and primary neurons in an expression-dependent manner (Fig. 2d, e). Transfection-mediated expression of poly-PR, the expression levels of which were comparable to those by the adenovirus-mediated expression (Fig. S5a), also caused the up-regulation of NEAT1 expression (Fig. S5b, c) as well as the neurotoxicity (Fig. S5d, e). In contrast, poly-GA did not appear to up-regulate the NEAT1 expression (Fig. 2b, c). Because of the insufficient expression of poly-GR and poly-PA, it could not be concluded whether they up-regulated the NEAT1 expression (Fig. 2b, c). Since a luciferase assay revealed that poly-PR markedly activated the *NEAT1* promoter (Fig. 2f), it is highly likely that poly-PR-caused increase in the NEAT1 expression occurs at the transcriptional level. In addition, we found that the poly-PR up-regulated exogenous NEAT1_1 (Fig. 2g, h) and exogenous NEAT1_2 (Fig. 2i, j). These results suggest that the poly-PR-mediated increase in the NEAT1 expression also occurs at the post-transcriptional level.

We then examined whether poly-PR-mediated up-regulation of NEAT1 and paraspeckles alters the expression of downstream genes. Consistent with a previous report^40^, poly-PR significantly increased the expression levels of NEAT1/paraspeckles-dependent gene, Nfkb1 and Nr4a1, in NSC-34 cells (Fig. 2k, l). Using HeLa cells in which poly-PR caused cytotoxicity and up-regulated the NEAT1 expression (Fig. 2m–o), we found that poly-PR up-regulated CCL5 and CXCL8, and down-regulated SH3PXD2A and TSHZ2, the expression of which are known to be positively or negatively regulated by NEAT1/paraspeckles, respectively^20,21^ (Fig. 2o). These results together suggest that NEAT1 and paraspeckles, the levels of which were up-regulated by poly-PR, are functional although we cannot exclude the possibility that poly-PR regulated paraspeckle-regulated gene expressions independently of NEAT1/paraspeckles.

### NEAT1 up-regulation causes neurotoxicity

**Fig. 3:**
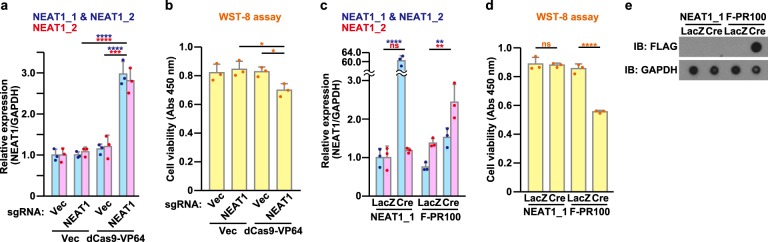
Up-regulation of NEAT1 causes neurotoxicity. **a**, **b** NSC-34 cells were transfected with 0.25 μg/well of the U6-sgRNA(MS2)_EF1a-MS2-P65-HSF1 vector (Vec) or the guide RNA sequence against *NEAT1* promoter-containing U6-sgRNA(MS2)_EF1a-MS2-P65-HSF1 vector (NEAT1) in association with 0.75 μg/well of the pcDNA3 vector (Vec) or the pAC152-dual-dCas9VP64-sgExpression vector (dCas9-VP64) on 6-well plate. At 72 h after the transfection, the quantitative real-time PCR analysis of NEAT1 was performed (**a**). The cell viability was detected by WST-8 assay (**b**). The data are presented as means ± SD (*N* = 3). Statistical analysis was performed by one-way ANOVA followed by the Tukey’s multi comparisons test. **c**–**e** NSC-34 cells were infected with adenovirus encoding mouse NEAT1_1 or FLAG-PR100 at an MOI of 400 together with adenovirus encoding LacZ or Cre-recombinase at an MOI of 40. At 48 h after the infection, the quantitative real-time PCR analysis of NEAT1 was performed (**c**). The cell viability was detected by WST-8 assay (**d**). The cell lysates were subjected to dot blotting analysis using indicated antibodies (**e)**. The data are presented as means ± SD (*N* = 3). Statistical analysis for data obtained from (**c**) NEAT1_2 and (**d**) was performed by one-way ANOVA followed by the Tukey’s multi comparisons test. Statistical analysis for data obtained from (**c**) NEAT1_1 & NEAT1_2 was conducted by the unpaired *t*-test

We then examined whether the increase in the expression of NEAT1 affected neuronal cell viability. The NEAT1 promoter activation mediated by CRISPR activation system^41^ resulted in an approximately 3-fold increase in the expression of NEAT1 compared to the basal level (Fig. 3a). Importantly, we observed that the up-regulation in the NEAT1 expression caused significant reduction in neuronal cell viability (Fig. 3b). Combined with the results that poly-PR up-regulated NEAT1 expression (Fig. 2), this result suggests that the poly-PR-induced neurotoxicity may be mediated by, at least in part, the up-regulation of NEAT1 expression.

To examine which variants of NEAT1 are responsible for reducing cell viability, we overexpressed NEAT1_1 by adenovirus gene transfer and found that robust overexpression of NEAT1_1 (Fig. 3c) did not cause any neurotoxicity (Fig. 3d, e). Unfortunately, a very long nucleotide sequence of NEAT1_2 disturbed the generation of the NEAT1_2-expressing recombinant virus. Thus, we were unable to address whether NEAT1_2 overexpression caused neurotoxicity. However, according to the results obtained in Fig. 3, we assume that NEAT1_2 is essential for neurotoxicity induced by NEAT1 up-regulation.

Moreover, we found that silencing of NEAT1 by 2 types of ASOs also reduced neuronal cell viability (Fig. S6) as shown in a previous study^40^. These data suggest that the cellular levels of NEAT1 may be tightly regulated within a narrow normal range and the deviation of the NEAT1 level from the normal range may result in neurotoxicity.

### HnRNPM may be involved in the poly-PR-induced neurotoxicity mediated by the up-regulation of NEAT1 expression

**Fig. 4:**
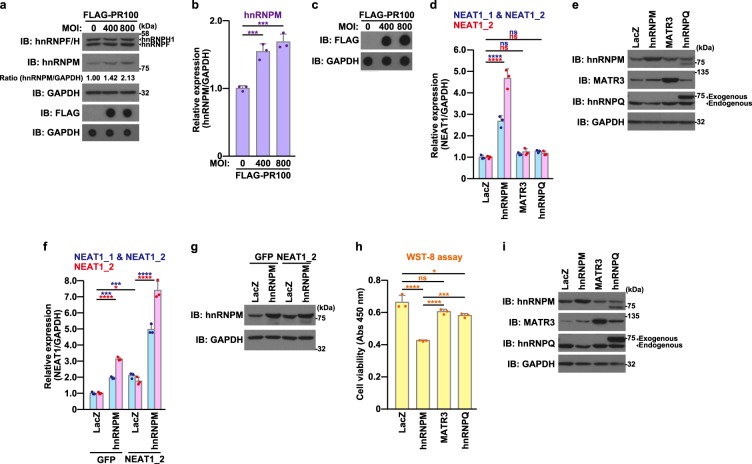
Overexpression of hnRNPM up-regulates NEAT1 expression and induces neurotoxicity. **a** NSC-34 cells were infected with adenovirus encoding FLAG-PR100 at MOIs of 0–800. To keep the constant total MOIs of adenoviruses, appropriate MOIs of LacZ-encoding adenovirus were added for each infection. At 48 h after the infection, the cell lysates were subjected to immunoblotting (IB) and dot blotting analysis using indicated antibodies. Intensities of immunodetected signals of endogenous hnRNPM were densitometrically examined with an ImageJ software. **b**, **c** NSC-34 cells were infected with adenovirus encoding FLAG-PR100 at MOIs of 0–800. To keep the constant total MOIs of adenoviruses, appropriate MOIs of LacZ-encoding adenovirus were added for each infection. At 48 h after the infection, the quantitative real-time PCR analysis of hnRNPM was performed (**b**). The cell lysates were subjected to dot blotting analysis using indicated antibodies (**c**). The data are presented as means ± SD (*N* = 3). Statistical analysis was performed by one-way ANOVA followed by the Dunnett’s multi comparisons test. **d**, **e** NSC-34 cells were infected with adenovirus encoding LacZ, hnRNPM, MATR3, or hnRNPQ at an MOI of 400. At 48 h after the infection, the quantitative real-time PCR analysis of NEAT1 was performed (**d**). The cell lysates were subjected to immunoblotting (IB) using indicated antibodies (**e**). The data are presented as means ± SD (*N* = 3). Statistical analysis was performed by one-way ANOVA followed by the Tukey’s multi comparisons test. **f**, **g** NSC-34 cells were transfected with 0.2 μg/well of the pCMV-GFP or -mouse NEAT1_2 on 6-well plate. After the transfection, NSC-34 cells were infected with adenovirus encoding LacZ or hnRNPM at an MOI of 400. At 48 h after the infection, the quantitative real-time PCR analysis of NEAT1 was performed (**f**). The cell lysates were subjected to immunoblotting (IB) using indicated antibodies (**g**). The data are presented as means ± SD (*N* = 3). Statistical analysis was performed by one-way ANOVA followed by the Dunnett’s multi comparisons test. **h**, **i** NSC-34 cells were infected with adenovirus encoding LacZ, hnRNPM, MATR3, or hnRNPQ at an MOI of 400. At 48 h after the infection, the cell viability was detected by WST-8 assay (**h**). The cell lysates were subjected to immunoblotting (IB) using indicated antibodies (**i**). The data are presented as means ± SD (*N* = 3). Statistical analysis was performed by one-way ANOVA followed by the Tukey’s multi comparisons test

It is hypothesized that the expression levels of poly-PR-binding paraspeckle hnRNPs are affected by poly-PR (Fig. 1). We found that poly-PR increased hnRNPM expression at both the protein and the mRNA level (Fig. 4a–c), but did not affect those of hnRNPF and hnRNPH1 (Fig. 4a). We then examined whether the increase in the hnRNPM expression affected the NEAT1 expression. The effect of another paraspeckle protein, MATR3^42^, and another hnRNPM-related protein belonging to the hnRNP family, hnRNPQ^43^, both of which bound to poly-PR (Fig. S2a), were examined as negative controls. The low-level overexpression of hnRNPM, but not that of MATR3 or hnRNPQ, up-regulated the NEAT1 expression (Fig. 4d, e). We then examined whether hnRNPM up-regulated NEAT1 expression transcriptionally, post-transcriptionally, or both. A luciferase assay showed that hnRNPM did not activate the *NEAT1* promoter at all (Fig. S7a, b). Although hnRNPM did not affect the level of exogenous NEAT1_1 (Fig. S7c, d), it increased the expression level of exogenous NEAT1_2 (Fig. 4f, g). These results indicate that hnRNPM specifically up-regulates the NEAT1_2 expression by stabilizing it. As expected, the overexpression of hnRNPM, the expression level of which was 1–2 times larger than the endogenous level, reduced neuronal cell viability, whereas that of MATR3 or hnRNPQ caused no or less neurotoxicity (Fig. 4h, i). These results together suggest that the neurotoxicity caused by the poly-PR-induced up-regulation of NEAT1 may be mediated by, at least in part, via the up-regulation of hnRNPM.

### Poly-PR suppresses the function of hnRNPF

**Fig. 5:**
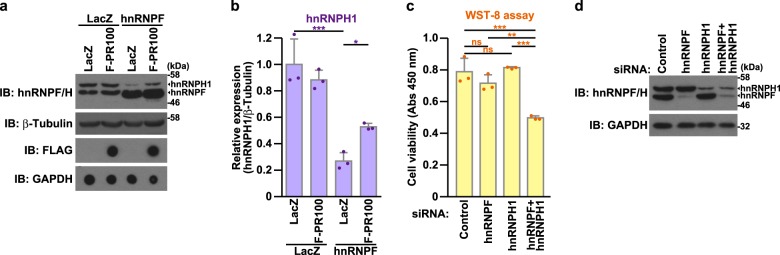
Poly-PR-induced suppression of hnRNPF/H1 function causes neurotoxicity. **a**, **b** NSC-34 cells were infected with adenovirus encoding LacZ or hnRNPF at an MOI of 200. Cells were also co-infected with adenovirus encoding LacZ or FLAG-PR100 at an MOI of 400. At 48 h after the infection, the cell lysates were subjected to immunoblotting (IB) and dot blotting analysis using indicated antibodies (**a**). Intensities of immunodetected signals of endogenous hnRNPH1 were densitometrically examined with an ImageJ software (**b**). The data are presented as means ± SD (*N* = 3). Statistical analysis was performed by one-way ANOVA followed by the Dunnett’s multi comparisons test. **c**, **d** NSC-34 cells were co-transfected with the 5 nM control, hnRNPF, and hnRNPH1 siRNAs. To keep the total concentration of siRNA at 10 nM, an appropriate amount of control siRNA was added for each transfection. At 60 h after the transfection, the cell viability was detected by WST-8 assay (**c**). The cell lysates were subjected to immunoblotting (IB) analysis using indicated antibodies (**d**). The data are presented as means ± SD (*N* = 3). Statistical analysis was performed by one-way ANOVA followed by the Tukey’s multi comparisons test

We next examined whether poly-PR affected the function of poly-PR-interacting paraspeckle-localizing hnRNPs, hnRNPF and hnRNPH1 (Fig. 1). We found that overexpression of hnRNPF, the expression level of which was 1–2 times larger than the endogenous level, significantly reduced the expression of endogenous hnRNPH1 (Fig. 5a, b, and S8a). Based on the facts that hnRNPF have a strong structural similarity to hnRNPH1^44^, they have been shown to co-immunoprecipitate^45^ and to have functional similarities^44,46–50^, it could be speculated that their function may be similar or redundant and the overexpression of one gene (hnRNPF) causes the so-called feedback reduction in the expression of the other gene (hnRNPH1). Using this expression-regulating system to monitor the function of hnRNPF, we investigated whether poly-PR affected the function of hnRNPF. As expected, we found that poly-PR attenuated the hnRNPF-mediated down-regulation of hnRNPH1 expression (Fig. 5a, b, and S8a), indicating that poly-PR inhibits the function of hnRNPF. Because poly-PR bound to both hnRNPF and hnRNPH1 similarly (Fig. 1), it could be assumed that poly-PR may also inhibit the function of hnRNPH1.

We then investigated whether the poly-PR-mediated inhibition of hnRNPF and hnRNPH1 function affected cell viability. While the siRNA-mediated silencing of hnRNPF or hnRNPH1 expression did not affect the cell viability (Fig. 5c, d), the reduction in both hnRNPF and hnRNPH1 expression significantly reduced the cell viability (Fig. 5c, d). The results suggest that poly-PR-caused neurotoxicity may be partially mediated by the down-regulation of hnRNPF and hnRNPH1. In addition, we found that the low-level overexpression of hnRNPF or hnRNPH1 reduced neuronal cell viability (Fig. S8b,c). These data suggest that the deviation in hnRNPF/H function causes neurotoxicity.

### The dysregulation in the TDP-43 expression up-regulates the expression of NEAT1

**Fig. 6:**
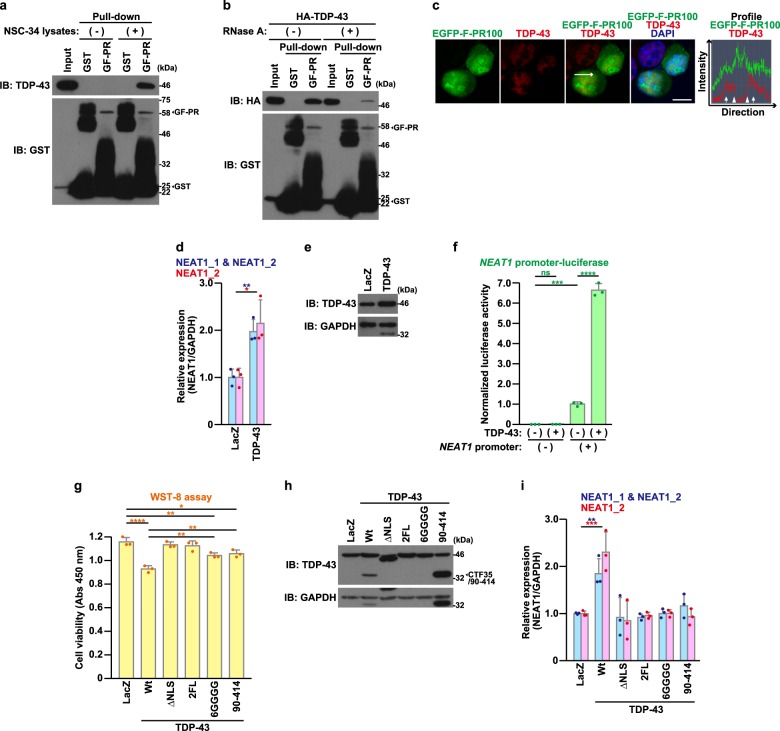
Poly-PR interacts and co-localizes with TDP-43 and nuclear TDP-43 up-regulates NEAT1 expression. **a** GST or GST-FLAG-PR100 (GF-PR)-bound glutathione beads were mixed with (+) or without (−) NSC-34 cell lysates. After rotation at 4 °C overnight, the glutathione beads were washed and were subjected to immunoblotting (IB) using indicated antibodies. In western blotting using anti-GST antibody, the large smear within the molecular weights ranging 25–46 kDa in the GF-PR lane is thought to consist of C-terminal truncated GST-FLAG-PR100 proteins. A band located around 50 kDa in the GST lane is thought to represent dimerized and/or aggregated GST-derived proteins. **b** Lysates of NSC-34 cells overexpressing HA-tagged TDP-43 and GST or GST-FLAG-PR100 (GF-PR)-bound glutathione beads were incubated with (+) or without (−) 20 μg/mL RNase A. After the incubation, the cell lysates were mixed with recombinant GST or GST-FLAG-PR100-bound glutathione beads. The glutathione beads were washed and were subjected to immunoblotting (IB) using indicated antibodies. **c** NSC-34 cells overexpressing EGFP-FLAG-PR100 (EGFP-F-PR100) (green) were fixed and were immunostained with TDP-43 antibody (red). Nuclei were stained with DAPI (blue). Scale bar = 10 μm. The rightmost panel shows the profile image of fluorescence intensities on the line of EGFP-FLAG-PR100 and TDP-43-merged image. Arrows and arrowheads indicate the localization of nucleoplasm-localizing EGFP-FLAG-PR100 and the border of nucleolus-localizing EGFP-FLAG-PR100, respectively. **d**, **e** NSC-34 cells were infected with adenovirus encoding LacZ or TDP-43 at an MOI of 800. At 48 h after the infection, the quantitative real-time PCR analysis of NEAT1 was performed (**d**). The cell lysates were subjected to immunoblotting (IB) using indicated antibodies (**e**). The data are presented as means ± SD (*N* = 3). Statistical analysis was performed by the unpaired t-test. **f** NSC-34 cells were transfected with the *NEAT1*-promoter (+) or *NEAT1*-promoterless (−) luciferase vector together with the pEF1-Myc/His-vec (−) or the pEF1-TDP-43 (+). At 48 h after the transfection, the luciferase activity was measured. The data are presented as means ± SD (*N* = 3). Statistical analysis was performed by one-way ANOVA followed by the Tukey’s multi comparisons test. **g**–**i** NSC-34 cells were infected with adenovirus encoding LacZ or TDP-43 derivatives at an MOI of 800. At 48 h after the infection, the cell viability was detected by WST-8 assay (**g**). The cell lysates were subjected to immunoblotting (IB) using indicated antibodies (**h**). CTF35, C-terminal fragment 35 kDa. The quantitative real-time PCR analysis of NEAT1 was performed (**i**). The data are presented as means ± SD (*N* = 3). Statistical analysis for (**g**) was performed by one-way ANOVA followed by the Tukey’s multi comparisons test. Statistical analysis for (**i**) was conducted by one-way ANOVA followed by the Dunnett’s multi comparisons test

Since C9-ALS/FTD has been known to be associated with the TDP-43 pathology^6^, it is likely that TDP-43 contributes to the pathogenesis of C9-ALS/FTD. As expected, poly-PR bound to not only exogenous HA-TDP-43, but also endogenous TDP-43 (Fig. 6a, b). The interaction was dependent on the presence of RNAs (Fig. 6b). The deletion of LCD of TDP-43 or another poly-PR-interacting paraspeckle protein RBM14 (Fig. S2a) diminished their interaction with poly-PR (Fig. S9), suggesting that their interaction with poly-PR is mediated through LCD. TDP-43 and poly-PR co-localized in the nucleoplasm (Fig. 6c and S3d), consistently with a previous study^51^. The increased paraspeckle formation and the up-regulation of NEAT1 expression are observed in affected regions in ALS/FTD patients and TDP-43 associates with NEAT1_2 RNA and some paraspeckle proteins^25–28,34,52,53^. Given the interaction between poly-PR and TDP-43 and the possible involvement of NEAT1 and paraspeckles in the poly-PR-caused neurotoxicity shown above, these reported findings prompted us to examine whether TDP-43 affected NEAT1 expression. We then found that the low-level overexpression of TDP-43, the expression level of which was 1–2 times larger than the endogenous level and was similar or less to the expression level that was observed in pathological conditions^54–58^, significantly up-regulated the expression of NEAT1 (Fig. 6d, e), at least partially, through the activation of the *NEAT1* promoter (Fig. 6f).

As demonstrated in our previous studies^33,34^, low-grade overexpression of TDP-43 significantly reduced neuronal cell viability (Fig. 6g, h). In contrast, other TDP-43 derivatives that lack a nuclear localization signal (ΔNLS), an RNA-binding (2FL), a self-dimerization signal (6GGGG), or the N-terminal portion of TDP-43 (Δ1–89 amino acid) showed no or less neurotoxicity compared with wild-type (wt) TDP-43 (Fig. 6g, h). Interestingly, any tested TDP-43 derivatives did not increase the expression of NEAT1 (Fig. 6i). These results indicate that the nuclear localization, the dimerization, and the RNA-binding of TDP-43, are necessary for the TDP-43-mediated increase in the NEAT1 level.

**Fig. 7:**
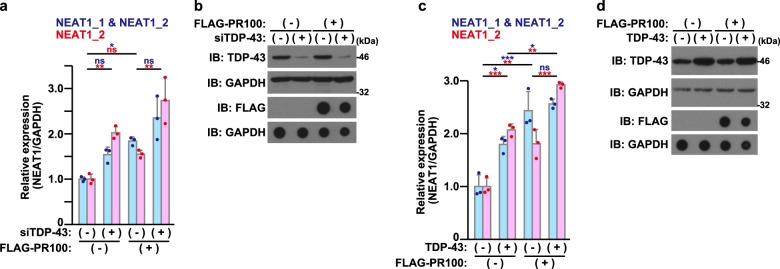
Poly-PR and TDP-43 up-regulate NEAT1 expression through distinct mechanisms. **a, b** NSC-34 cells were transfected with the 5 nM control siRNA (−) or the TDP-43 siRNA (+). At 18 h after the transfection, cells were infected with adenovirus encoding LacZ (−) or FLAG-PR100 (+) at an MOI of 400. At 48 h after the infection, the quantitative real-time PCR analysis of NEAT1 was performed (**a**). The cell lysates were subjected to immunoblotting (IB) and dot blotting analysis using indicated antibodies (**b**). The data are presented as means ± SD (*N* = 3). Statistical analysis was performed by one-way ANOVA followed by the Tukey’s multi comparisons test. **c**, **d** NSC-34 cells were infected with adenovirus encoding LacZ (−) or TDP-43 (+) at an MOI of 400. Cells were also co-infected with adenovirus encoding LacZ (−) or FLAG-PR100 (+) at an MOI of 200. At 48 h after the infection, the quantitative real-time PCR analysis of NEAT1 was performed (**c**). The cell lysates were subjected to immunoblotting (IB) and dot blotting analysis using indicated antibodies (**d**). The data are presented as means ± SD (*N* = 3). Statistical analysis was performed by one-way ANOVA followed by the Tukey’s multi comparisons test

Given that poly-PR binds to TDP-43 (Fig. 6a, b) and that TDP-43 up-regulates NEAT1 expression (Fig. 6d, e), it could be assumed that the poly-PR-induced up-regulation of the NEAT1 expression was mediated by TDP-43. To address this possibility, we examined whether the knock-down of the TDP-43 expression attenuated the poly-PR-caused up-regulation of the NEAT1 expression. However, the knock-down of the TDP-43 expression up-regulated the NEAT1_2 expression by itself (Fig. 7a, b), consistently with a previous report^26^. Furthermore, the poly-PR expression and the knock-down of the TDP-43 expression up-regulated the NEAT1_2 expression in a seemingly additive manner (Fig. 7a, b). We also found that co-expression of poly-PR and TDP-43 additively up-regulated the expression of NEAT1_2 (Fig. 7c, d).

## Discussion

In this study, we have shown that poly-PR up-regulates the expression of NEAT1 (Fig. 2). The CRISPR-assisted up-regulation of NEAT1 results in the reduction of neuronal cell viability (Fig. 3). These results suggest that poly-PR may cause neurotoxicity, at least in part, by the up-regulation of NEAT1 expression. To obtain the direct evidence supporting this hypothesis, we attempted to demonstrate that the knock-down of endogenous NEAT1 attenuated the poly-PR-caused neurotoxicity. However, we encountered the fact that the reduction in the expression of NEAT1 also caused neurotoxicity by itself (Fig. S6). Consequently, we could not examine the involvement of NEAT1 in the poly-PR-induced neurotoxicity in a direct manner.

The mechanism underlying the poly-PR-mediated up-regulation of NEAT1 remains speculative. Given that poly-PR increases the expression of hnRNPM (Fig. 4a–c) and that the overexpression of hnRNPM up-regulates the NEAT1 expression by specifically stabilizing NEAT1_2 (Fig. 4d–g), it is likely that hnRNPM is the downstream mediator in the regulation of the poly-PR-mediated NEAT1_2 up-regulation. It is also possible that poly-PR up-regulates the expression of NEAT1 through undetermined multiple pathways besides the mechanism we identified.

The RIP assay demonstrated that poly-PR interacted with NEAT1 (Fig. 1d, e). In agreement, RNA FISH analysis showed the partial co-localization between poly-PR and NEAT1 (Fig. 1f). It has been shown that NEAT1 is mainly localized in the paraspeckles and also in the non-paraspeckle foci, called microspeckels^59,60^, in the nucleoplasm, and poly-PR is localized mainly in the nucleolus and minorly in the nucleoplasm^10^. Thus, it could be assumed that nucleoplasm-localizing poly-PR mainly contributes to the interaction with NEAT1. Furthermore, it is possible that poly-PR interacts with NEAT1 indirectly through the paraspeckle proteins such as hnRNPs, SFPQ, NONO, and TDP-43 (Figs 1 and 6).

The mechanism underlying the NEAT1-mediated neurotoxicity remains unknown. Multiple studies reported that the level of the NEAT1 expression is up-regulated in patients with numerous neurodegenerative diseases^24^. However, it remains controversial that NEAT1 displays neuroprotective^40,61^ or detrimental^62,63^ effect on neurodegeneration. Further studies are needed to clarify the pathological roles of NEAT1/paraspeckle in neurodegeneration.

NEAT1_2, but not NEAT1_1, was probably necessary for the induction of neurotoxicity (Fig. 3c–e). Given that NEAT1_2 is essential for the paraspeckle formation^11^, it could be assumed that the increase in the paraspeckle formation, caused by the up-regulation of NEAT1_2, is essential for the induction of neurotoxicity. Since paraspeckles have been known to participate in RNA metabolism^18–23^, it is likely that the poly-PR-mediated increase in the paraspeckle formation leads to the dysregulation of downstream RNA metabolism and induces neurotoxicity. In agreement, we found that poly-PR altered the expression levels of paraspeckle-dependent genes (Fig. 2k–o). Given that NEAT1_1 also localizes in the non-paraspeckle foci, microspeckles, that may have paraspeckle-unrelated functions^60^, it is also possible that poly-PR causes neurotoxicity by modulating NEAT1_1 in non-paraspeckle cellular compartments^27,60^.

Emerging evidence suggests that paraspeckles are formed by the nuclear liquid-liquid phase separation (LLPS) that is mediated by RNA molecules and RNA-binding proteins with a prion-like LCD, an intrinsically disordered domain^29,64,65^. It has been reported that aberrant LLPS, caused by mutations in the LCDs of some RNA-binding proteins or persistent stress, accelerates the conversion of membrane-less organelles including paraspeckles from physiological liquid-like droplet or hydrogel-like states to the state of fibrillization, resulting in the formation of pathological irreversible amyloid-like aggregates^66–68^. Given that poly-PR binds to NEAT1 as well as multiple proteins that possess LCDs^51^ including TDP-43 and RBM14 probably through their LCDs (Fig. S9), it could be hypothesized that poly-PR may increase paraspeckle formation and accelerate the formation of toxic aggregates in the nucleus by modulating the function of the LLPS-regulating paraspeckle proteins.

We recognized the interaction between poly-PR and other lncRNAs than NEAT1 (Fig. S2e). This result could be expected to occur since poly-PR has been known to bind to an RNA backbone structure^39^. If this is the case, poly-PR non-specifically binds to RNA. It is also possible that poly-PR binds to these lncRNAs through a common conformational domain of lncRNA or through some intermediate proteins that bind to both lncRNAs^52^ and poly-PR. In any event, these observations suggest that there may be other lncRNA targets of poly-PR than NEAT1 and the mechanism underlying the poly-PR-mediated neurotoxicity may be more diverse.

Multiple clinical studies have demonstrated that the levels of TDP-43 expression are up-regulated in sporadic ALS^54–58^. We have previously shown that the low-level overexpression of nucleus-localizing full-length TDP-43 causes neuronal cell death and that the cleaved forms of TDP-43 such as CTF(C-terminal fragment)35, which accumulates in the cytoplasm in affected neurons in the patients with ALS/FTD^69,70^, have no or less neuronal toxicity^33,34^. A cleavage-resistant mutant of TDP-43 or the inhibition of TDP-43 fragmentation has stronger cell death-inducing activity than wt-TDP-43^33,71^. These findings suggest that the gain-of-toxic function of full-length nuclear TDP-43, but not cytoplasmic cleaved TDP-43, causes neurotoxicity. In this study, we have shown that nucleus-localizing full-length TDP-43, but not cytoplasmic and cleaved TDP-43, up-regulates the expression of NEAT1 (Fig. 6). NEAT1_2 expression and paraspeckle formation appears to occur preferentially at the early stage of ALS when the TDP-43 pathology is not generated^25^. Therefore, it is likely that nucleus-localizing full-length TDP-43 causes neurotoxicity by up-regulating NEAT1 expression in the early stages of ALS.

In the current study, we showed that the knock-down of the TDP-43 expression up-regulated the NEAT1_2 expression (Fig. 7a, b), as shown in the previous report^26^. The TDP-43 level may be strictly regulated within a narrow normal range and the dysregulation in the TDP-43 expression up-regulates NEAT1 expression (Fig. 7). Thus, both of the gain-of-function and the loss-of-function of TDP-43 may cause neurotoxicity by up-regulating NEAT1 expression. Since poly-PR and the dysregulation of TDP-43 independently up-regulate the expression of NEAT1_2 that is essential for the paraspeckle formation^11^ and probably for the induction of neurotoxicity (Figs. 3 and 7), it could be assumed that the dysregulation of TDP-43 also enhances the paraspeckle formation in ALS/FTD cases without C9ORF72 mutation.

In summary, the current study shows that poly-PR up-regulates the expression of NEAT1. The increase in NEAT1 expression by the CRISPR activation system in cultured neuronal cells reduces cell viability. This result suggests that the NEAT1 up-regulation may be involved in the poly-PR-mediated neurotoxicity. Poly-PR also binds to several paraspeckle proteins and modulates their functions. Furthermore, the alteration in the TDP-43 expression causes the NEAT1 up-regulation. These results together suggest that poly-PR and TDP-43 cause ALS/FTD-related neurotoxicity, at least in part, by increasing the NEAT1 expression and paraspeckle formation and by dysregulating the paraspeckle-localizing hnRNPs. Given that the C9ORF72 mutation is the most frequent genetic cause of ALS/FTD and that the majority of ALS patients exhibits the TDP-43 pathology, it could be hypothesized that the enhancement of the paraspeckle formation may contribute to ALS/FTD-related neurodegeneration as a central common neurodegenerative pathway. Further investigation on the molecular mechanism underlying the poly-PR neurotoxicity is required to uncover the C9ORF72-linked neurodegeneration.

## Supplementary information

Supplementary information

## References

[1] Ling SC, Polymenidou M, Cleveland DW (2013). Converging mechanisms in ALS and FTD: disrupted RNA and protein homeostasis. Neuron.

[2] Taylor JP, Brown RH, Cleveland DW (2016). Decoding ALS: from genes to mechanism. Nature.

[3] Rademakers R, Neumann M, Mackenzie IR (2012). Advances in understanding the molecular basis of frontotemporal dementia. Nat. Rev. Neurol..

[4] Pottier C, Ravenscroft TA, Sanchez-Contreras M, Rademakers R (2016). Genetics of FTLD: overview and what else we can expect from genetic studies. J. Neurochem..

[5] Gao FB, Almeida S, Lopez-Gonzalez R (2017). Dysregulated molecular pathways in amyotrophic lateral sclerosis-frontotemporal dementia spectrum disorder. EMBO J..

[6] DeJesus-Hernandez M (2011). Expanded GGGGCC hexanucleotide repeat in noncoding region of C9ORF72 causes chromosome 9p-linked FTD and ALS. Neuron.

[7] Renton AE (2011). A hexanucleotide repeat expansion in C9ORF72 is the cause of chromosome 9p21-linked ALS-FTD. Neuron.

[8] Cleary JD, Pattamatta A, Ranum LPW (2018). Repeat-associated non-ATG (RAN) translation. J. Biol. Chem..

[9] Freibaum BD, Taylor JP (2017). The role of dipeptide repeats in C9ORF72-related ALS-FTD. Front. Mol. Neurosci..

[10] Suzuki H, Shibagaki Y, Hattori S, Matsuoka M (2018). The proline-arginine repeat protein linked to C9-ALS/FTD causes neuronal toxicity by inhibiting the DEAD-box RNA helicase-mediated ribosome biogenesis. Cell Death Dis..

[11] Sasaki YT, Ideue T, Sano M, Mituyama T, Hirose T (2009). MENepsilon/beta noncoding RNAs are essential for structural integrity of nuclear paraspeckles. Proc. Natl Acad. Sci. USA.

[12] Sunwoo H (2009). MEN epsilon/beta nuclear-retained non-coding RNAs are up-regulated upon muscle differentiation and are essential components of paraspeckles. Genome Res..

[13] Yamazaki T (2018). Functional domains of NEAT1 architectural lncRNA induce paraspeckle assembly through phase separation. Mol. Cell.

[14] Naganuma T (2012). Alternative 3’-end processing of long noncoding RNA initiates construction of nuclear paraspeckles. EMBO J..

[15] Fong KW (2013). Whole-genome screening identifies proteins localized to distinct nuclear bodies. J. Cell Biol..

[16] Yamazaki T, Hirose T (2015). The building process of the functional paraspeckle with long non-coding RNAs. Front Biosci..

[17] Nakagawa, S., Yamazaki, T. & Hirose, T. Molecular dissection of nuclear paraspeckles: towards understanding the emerging world of the RNP milieu. *Open Biol*. **8**, 180150 (2018). 10.1098/rsob.180150 PMC6223218 30355755

[18] Prasanth KV (2005). Regulating gene expression through RNA nuclear retention. Cell.

[19] Chen LL, Carmichael GG (2009). Altered nuclear retention of mRNAs containing inverted repeats in human embryonic stem cells: functional role of a nuclear noncoding RNA. Mol. Cell.

[20] Hirose T (2014). NEAT1 long noncoding RNA regulates transcription via protein sequestration within subnuclear bodies. Mol. Biol. Cell.

[21] Imamura K (2014). Long noncoding RNA NEAT1-dependent SFPQ relocation from promoter region to paraspeckle mediates IL8 expression upon immune stimuli. Mol. Cell.

[22] Bottini S (2017). Post-transcriptional gene silencing mediated by microRNAs is controlled by nucleoplasmic Sfpq. Nat. Commun..

[23] Jiang L (2017). NEAT1 scaffolds RNA-binding proteins and the Microprocessor to globally enhance pri-miRNA processing. Nat. Struct. Mol. Biol..

[24] An H, Williams NG, Shelkovnikova TA (2018). NEAT1 and paraspeckles in neurodegenerative diseases: a missing lnc found?. Noncoding RNA Res..

[25] Nishimoto Y (2013). The long non-coding RNA nuclear-enriched abundant transcript 1_2 induces paraspeckle formation in the motor neuron during the early phase of amyotrophic lateral sclerosis. Mol. Brain.

[26] Shelkovnikova TA (2018). Protective paraspeckle hyper-assembly downstream of TDP-43 loss of function in amyotrophic lateral sclerosis. Mol. Neurodegener..

[27] An H (2019). ALS-linked FUS mutations confer loss and gain of function in the nucleus by promoting excessive formation of dysfunctional paraspeckles. Acta Neuropathol. Commun..

[28] Tsuiji H (2013). Spliceosome integrity is defective in the motor neuron diseases ALS and SMA. EMBO Mol. Med..

[29] Hennig S (2015). Prion-like domains in RNA binding proteins are essential for building subnuclear paraspeckles. J. Cell. Biol..

[30] Salton M (2011). Matrin 3 binds and stabilizes mRNA. PLoS ONE.

[31] Cheng AW (2013). Multiplexed activation of endogenous genes by CRISPR-on, an RNA-guided transcriptional activator system. Cell Res..

[32] Kulcsar PI (2017). Crossing enhanced and high fidelity SpCas9 nucleases to optimize specificity and cleavage. Genome Biol..

[33] Suzuki H, Lee K, Matsuoka M (2011). TDP-43-induced death is associated with altered regulation of BIM and Bcl-xL and attenuated by caspase-mediated TDP-43 cleavage. J. Biol. Chem..

[34] Suzuki H, Shibagaki Y, Hattori S, Matsuoka M (2015). Nuclear TDP-43 causes neuronal toxicity by escaping from the inhibitory regulation by hnRNPs. Hum. Mol. Genet..

[35] Eksioglu YZ (1994). Human neuroblastoma growth inhibitory factor (h-NGIF), derived from human astrocytoma conditioned medium, has neurotrophic properties. Brain Res..

[36] Sudo H (2000). Antibody-regulated neurotoxic function of cell-surface beta-amyloid precursor protein. Mol. Cell. Neurosci..

[37] Banker GA, Cowan WM (1977). Rat hippocampal neurons in dispersed cell culture. Brain Res..

[38] Marko M, Leichter M, Patrinou-Georgoula M, Guialis A (2010). hnRNP M interacts with PSF and p54(nrb) and co-localizes within defined nuclear structures. Exp. Cell Res..

[39] Kanekura K (2018). Characterization of membrane penetration and cytotoxicity of C9orf72-encoding arginine-rich dipeptides. Sci. Rep..

[40] Cheng C (2018). The long noncoding RNA NEAT1 is elevated in polyglutamine repeat expansion diseases and protects from disease-gene dependent toxicities. Hum. Mol. Genet..

[41] Konermann S (2015). Genome-scale transcriptional activation by an engineered CRISPR-Cas9 complex. Nature.

[42] Banerjee A, Vest KE, Pavlath GK, Corbett AH (2017). Nuclear poly(A) binding protein 1 (PABPN1) and Matrin3 interact in muscle cells and regulate RNA processing. Nucleic Acids Res..

[43] Geuens T, Bouhy D, Timmerman V (2016). The hnRNP family: insights into their role in health and disease. Hum. Genet..

[44] Mauger DM, Lin C, Garcia-Blanco M (2008). A. hnRNP H and hnRNP F complex with Fox2 to silence fibroblast growth factor receptor 2 exon IIIc. Mol. Cell. Biol..

[45] Chou MY, Rooke N, Turck CW, Black DL (1999). hnRNP H is a component of a splicing enhancer complex that activates a c-src alternative exon in neuronal cells. Mol. Cell. Biol..

[46] Caputi M, Zahler AM (2001). Determination of the RNA binding specificity of the heterogeneous nuclear ribonucleoprotein (hnRNP) H/H’/F/2H9 family. J. Biol. Chem..

[47] Black DL (2003). Mechanisms of alternative pre-messenger RNA splicing. Annu. Rev. Biochem..

[48] Martinez-Contreras R (2006). Intronic binding sites for hnRNP A/B and hnRNP F/H proteins stimulate pre-mRNA splicing. PLoS Biol..

[49] Du J, Wang Q, Ziegler SF, Zhou B (2018). FOXP3 interacts with hnRNPF to modulate pre-mRNA alternative splicing. J. Biol. Chem..

[50] Yamazaki T (2018). TCF3 alternative splicing controlled by hnRNP H/F regulates E-cadherin expression and hESC pluripotency. Genes Dev..

[51] Lee KH (2016). C9orf72 dipeptide repeats impair the assembly, dynamics, and function of membrane-less organelles. Cell.

[52] Tollervey JR (2011). Characterizing the RNA targets and position-dependent splicing regulation by TDP-43. Nat. Neurosci..

[53] Lagier-Tourenne C (2012). Divergent roles of ALS-linked proteins FUS/TLS and TDP-43 intersect in processing long pre-mRNAs. Nat. Neurosci..

[54] Steinacker P (2008). TDP-43 in cerebrospinal fluid of patients with frontotemporal lobar degeneration and amyotrophic lateral sclerosis. Arch. Neurol..

[55] Swarup V (2011). Deregulation of TDP-43 in amyotrophic lateral sclerosis triggers nuclear factor kappaB-mediated pathogenic pathways. J. Exp. Med..

[56] Verstraete E (2012). TDP-43 plasma levels are higher in amyotrophic lateral sclerosis. Amyotroph. Lateral Scler..

[57] Kasai T (2009). Increased TDP-43 protein in cerebrospinal fluid of patients with amyotrophic lateral sclerosis. Acta Neuropathol..

[58] Suzuki M (2010). Increased expression of TDP-43 in the skin of amyotrophic lateral sclerosis. Acta Neurol. Scand..

[59] Nakagawa S, Naganuma T, Shioi G, Hirose T (2011). Paraspeckles are subpopulation-specific nuclear bodies that are not essential in mice. J. Cell. Biol..

[60] Li R, Harvey AR, Hodgetts SI, Fox AH (2017). Functional dissection of NEAT1 using genome editing reveals substantial localization of the NEAT1_1 isoform outside paraspeckles. RNA.

[61] Sunwoo JS (2017). Altered expression of the long noncoding RNA NEAT1 in Huntington’s disease. Mol. Neurobiol..

[62] Yan W, Chen ZY, Chen JQ, Chen HM (2018). LncRNA NEAT1 promotes autophagy in MPTP-induced Parkinson’s disease through stabilizing PINK1 protein. Biochem. Biophys. Res. Commun..

[63] Liu Y, Lu Z (2018). Long non-coding RNA NEAT1 mediates the toxic of Parkinson’s disease induced by MPTP/MPP+ via regulation of gene expression. Clin. Exp. Pharmacol. Physiol..

[64] Lin Y, Protter DS, Rosen MK, Parker R (2015). Formation and maturation of phase-separated liquid droplets by RNA-binding proteins. Mol. Cell.

[65] Shin, Y. & Brangwynne, C. P. Liquid phase condensation in cell physiology and disease. *Science* **357**, eaaf4382 (2017). 10.1126/science.aaf4382 28935776

[66] Patel A (2015). A liquid-to-solid phase transition of the ALS protein FUS accelerated by disease mutation. Cell.

[67] Molliex A (2015). Phase separation by low complexity domains promotes stress granule assembly and drives pathological fibrillization. Cell.

[68] Murakami T (2015). ALS/FTD mutation-induced phase transition of FUS liquid droplets and reversible hydrogels into irreversible hydrogels impairs RNP granule function. Neuron.

[69] Arai T (2006). TDP-43 is a component of ubiquitin-positive tau-negative inclusions in frontotemporal lobar degeneration and amyotrophic lateral sclerosis. Biochem. Biophys. Res. Commun..

[70] Neumann M (2006). Ubiquitinated TDP-43 in frontotemporal lobar degeneration and amyotrophic lateral sclerosis. Science.

[71] Li Q, Yokoshi M, Okada H, Kawahara Y (2015). The cleavage pattern of TDP-43 determines its rate of clearance and cytotoxicity. Nat. Commun..

