# Supplementary material for: C9-ALS/FTD-linked proline–arginine dipeptide repeat protein associates with paraspeckle components and increases paraspeckle formation
Source: Cell Death Dis. 2019 Oct 3;10(10):746. doi: 10.1038/s41419-019-1983-5 (PMC6776546; doi:10.1038/s41419-019-1983-5)
Supplement: Supplementary file 1 — Supplementary information [file 41419_2019_1983_MOESM1_ESM.pdf]

Supplementary Information

Fig. S1 Absorbance at 450 nm in WST-8 cell viability assays is correlated with the number of cells.

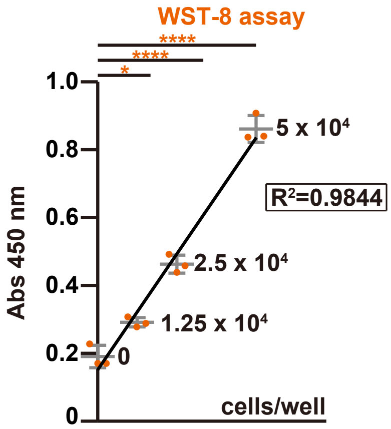

NSC-34 cells were seeded on 12-well plates at 1.25 × 10^4, 2.5 × 10^4, or 5 × 10^4 cells/well in DMEM supplemented with 10% FBS. At 48 h after the seeding, the cell viability was detected by the WST-8 assay. The data are presented as means ± SD (N = 3). Statistical analysis was performed by one-way ANOVA followed by the Tukey's multi comparisons test. A correlation coefficient is shown as R^2.

Fig. S2 Poly-PR associates with paraspeckle proteins.

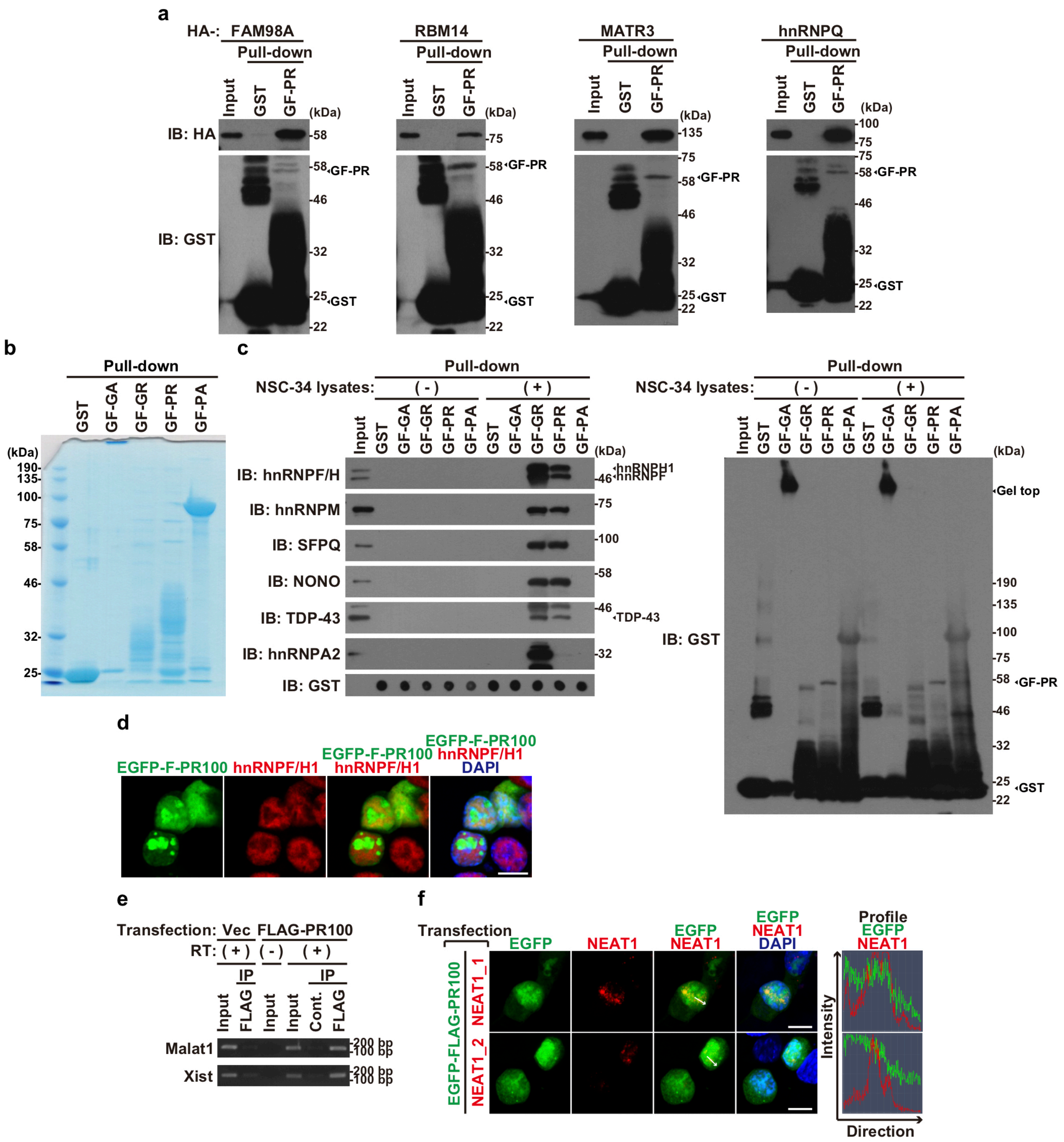

a Lysates of NSC-34 cells overexpressing HA-tagged FAM98A, RBM14, MATR3, or hnRNPQ were mixed with purified recombinant GST or GST-FLAG-PR100 (GF-PR)-bound glutathione beads. After rotation at 4 ºC, the glutathione beads were washed and were subjected to immunoblotting (IB) using indicated antibodies. In western blotting using anti-GST antibody, the large smear within the molecular weights ranging 25–46 kDa in the GF-PR lane is thought to consist of C-terminal truncated GST-FLAG-PR100 proteins. A band located around 50 kDa in the GST lane is thought to represent dimerized and/or aggregated GST-derived proteins.

b Recombinant GST-FLAG (GF)-DPR100 proteins used for pull-down binding assays were subjected to SDS-PAGE followed by Coomassie Brilliant Blue G staining. GST-FLAG-GA100 (GF-GA) showed a band at the gel top and a band with a molecular weight of 25 kDa (lane 2). GST-FLAG-GR100 (GF-GR) revealed smeared bands with molecular weights of 25–40 kDa and a band with a molecular weight of 58 kDa (lane 3). GST-FLAG-PR100 (GF-PR) generated smeared bands with molecular weights of 25–50 kDa and a band with a molecular weight of 58 kDa (lane 4). GST-FLAG-PA100 (GF-PA) showed a prominent band with molecular weights of 80–90 kDa (lane 5).

c GST or GST-FLAG-DPR100 (GF-GA, GR, PR, or PA)-bound glutathione beads were mixed with (+) or without (-) NSC-34 cell lysates. After the rotation at 4 ºC overnight, the glutathione beads were washed and were subjected to 5–20% gradient gel SDS-PAGE followed by immunoblotting (IB) or dot blotting analysis using indicated antibodies.

d NSC-34 cells overexpressing EGFP-FLAG-PR100 (EGFP-F-PR100) (green) were fixed and immunostained with hnRNPF/H antibody (red). Nuclei were stained with DAPI (blue). Scale bar = 10 μm.

e NSC-34 cells were transfected with the empty vector or the FLAG-PR100-encoding vector. At 48 h after the transfection, the cell lysates were immunoprecipitated (IP) with normal mouse IgG1 (Cont.) or the FLAG antibody. Precipitates were then used for RNA immunoprecipitation (RIP) assays. Reverse transcription (RT) (-) was used as negative control to monitor the PCR amplification from genomic DNA.

f NSC-34 cells overexpressing EGFP-FLAG-PR100 (green) together with mouse NEAT1_1 or NEAT1_2 were fixed and stained with the 125 nM Quasar 570-labeled NEAT1 Stellaris probe (red). Nuclei were stained with DAPI (blue). Scale bar = 10 μm. The rightmost panel shows the profile image of fluorescence intensities on the lines of EGFP-FLAG-PR100 and NEAT1-merged image.

Fig. S3 Poly-PR partially co-localizes with hnRNPs and TDP-43.

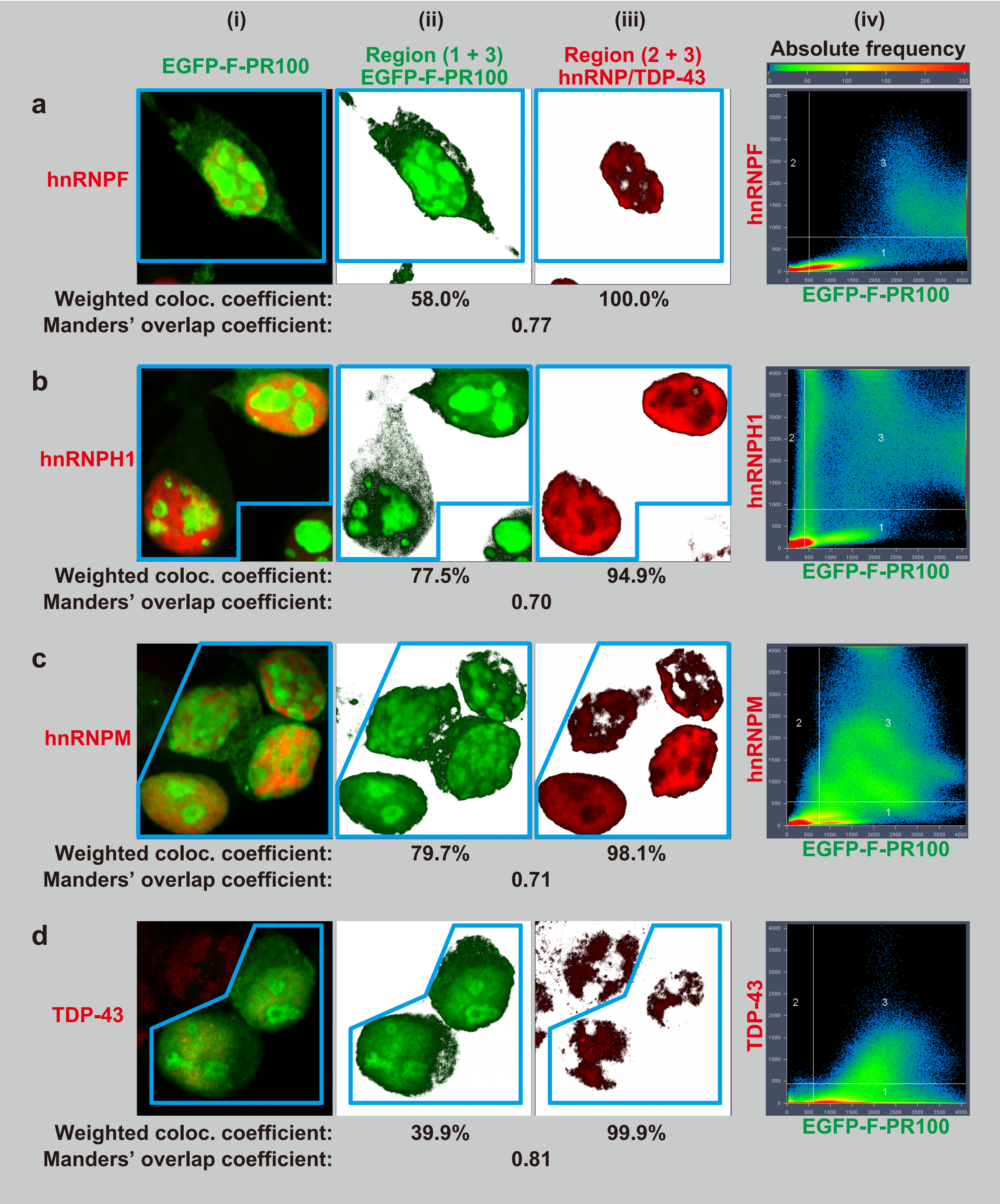

a-d The merged images in the panel (i) are the same as those in Figure 1b for hnRNPs or Figure 6c for TDP-43. The boxed regions surrounded by light blue lines, in which the cells express both proteins, EGFP-FLAG-PR100 (EGFP-F-PR100) and hnRNPF (a), hnRNPH1(b), hnRNPM (c), or TDP-43 (d), were analyzed by co-localization analysis by ZEN2010 software. The green color derived from EGFP-F-PR100 in the panel (ii) and the red color derived from hnRNPs or TDP-43 in the panel (iii) are corresponding to the regions that have the fluorescence intensity above the threshold line in the panel (iv) (Region 1+3 in the panel (iv) for EGFP-F-PR100 and Region 2+3 in the panel (iv) for hnRNPs or TDP-43). The white color in the panel (ii) and (iii) are corresponding to the regions that have the fluorescence intensity below the threshold line in the panel (iv). The panel (iv) represents the scattergram consisting of the fluorescence intensity of EGFP-F-PR100 (X-axis) and that of hnRNPs or TDP-43 (Y-axis). The absolute frequency is shown as gradational color. The weighted colocalization coefficient (weighted coloc. coefficient) and the Manders' overlap coefficient were calculated based on the scattergram shown in the panel (iv) and were shown under the panel (ii) and (iii). The weighted colocalization coefficient indicates the co-localizing fluorescence intensities relative to the total fluorescence intensities above the background. The values for the Manders' overlap coefficient range from 0 to 1 and a value of 1 shows perfect co-localization (Ref. Manders, E.M.M., Verbeek, F.J., & Aten, J.A. Measurement of co-localization of objects in dual-colour confocal images. *J Microsc* **169**, 375–382 (1993)).

Fig. S4 Poly-PR increases paraspeckle formation.

a

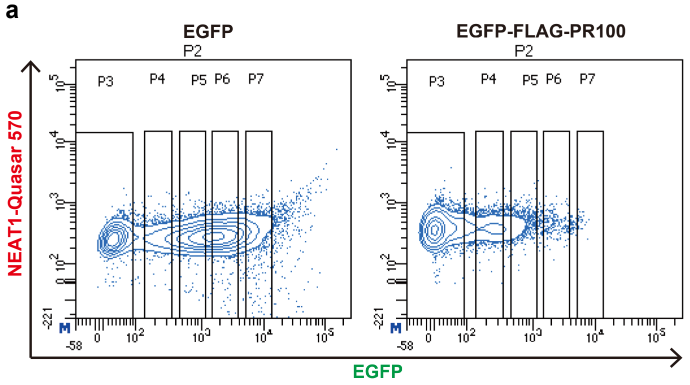

b

Population	EGFP: EGFP mean	EGFP: NEAT1 mean	EGFP-FLAG-PR100: EGFP mean	EGFP-FLAG-PR100: NEAT1 mean
P3	38	334	18	463
P4	241	346	231	482
P5	798	351	680	536
P6	2,413	373	2,410	530
P7	7,874	400	5,782	566

c

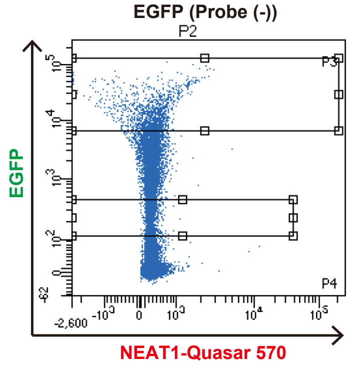

d

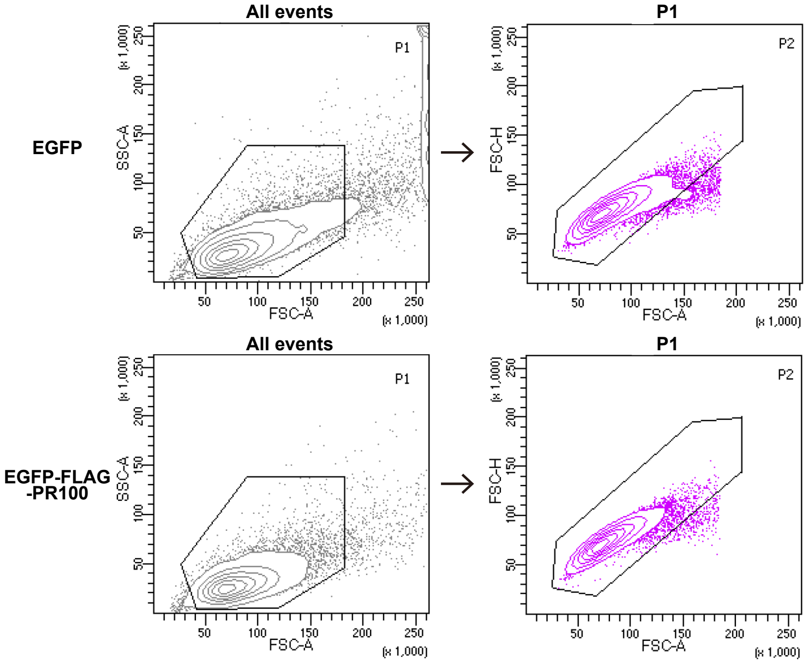

a, b, c NSC-34 cells transfected with EGFP or EGFP-FLAG-PR100 were fixed and stained with 1000 nM Quasar 570-conjugated Stellaris probe that hybridizes the mouse 5’ segment of NEAT1 RNA under the suspension condition. Fluorescence was analyzed with flow cytometry (a). The mean of fluorescence intensity of each population (P3-P7) in (a) is shown in (b). Note that false positive Quasar 570 fluorescence signals are observed in the EGFP-high region in EGFP-expressing cells due to the correction effect of overloaded signals of EGFP. The same signals are also observed in EGFP-expressing cells that were not stained with FISH probe (c, P3).

d Gating strategies for the flow cytometry are shown. The cells were primarily gated to remove debris (left panel, FSC/SSC, population 1 [P1]). The cells in P1 were further gated for singlet cells (right panel, FSC-H/FSC-A, population 2 [P2]). Then, the cells in P2 were analyzed for fluorescence analysis as shown in (a).

Fig. S5 Poly-PR, expressed by plasmid transfection and adenovirus infection, causes neurotoxicity and the up-regulation of NEAT1 expression.

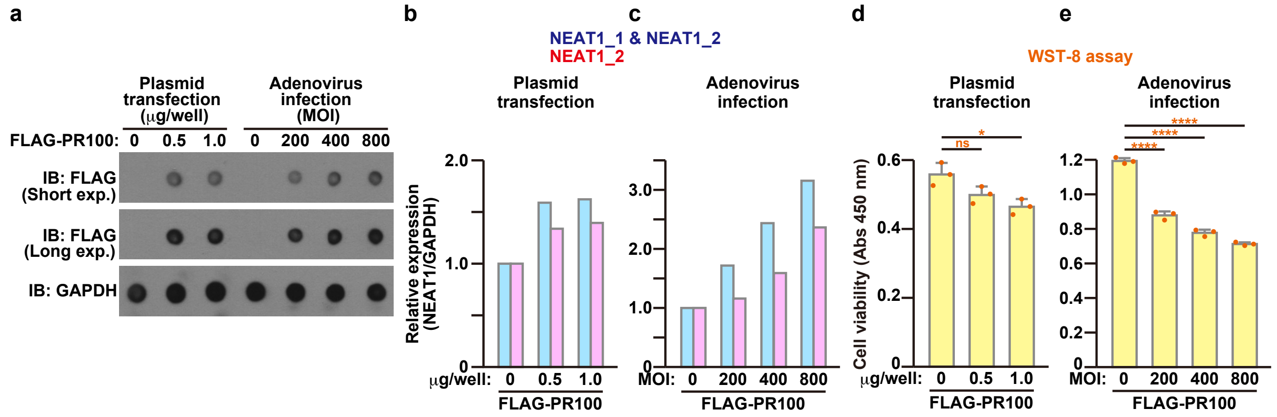

a-e NSC-34 cells were transfected with 0.5-1.0 μg/well of the pEF1-FLAG-PR100 on 6-well plates. To keep the constant total amount of plasmid, an appropriate amount of the pEF1-Myc/His-vec (the backbone vector) was added for each transfection (a, b, d). Separately, NSC-34 cells were infected with adenovirus encoding FLAG-PR100 at MOIs of 0–800. To keep the constant total MOIs of adenoviruses, appropriate MOIs of LacZ-encoding adenovirus were added for each infection (a, c, e). At 48 h after the transfection or infection, the cell lysates were subjected to dot blotting analysis using indicated antibodies (exp, exposure) (a). The quantitative real-time PCR analysis of NEAT1 was performed (b, c) and the cell viability was detected by WST-8 assay (d, e). The data are presented as means ± SD (N = 3) and statistical analysis was performed by one-way ANOVA followed by the Dunnett's multi comparisons test (d, e). The non-specific cytotoxicity accompanied with transfection itself reduced the basal cell viability in transfection samples, as compared with that in adenovirus-infected samples (compare Abs 450 nm Y-axis of plasmid transfection (d) with that of adenovirus infection (e)).

Fig. S6 Knock-down of NEAT1 causes the neurotoxicity.

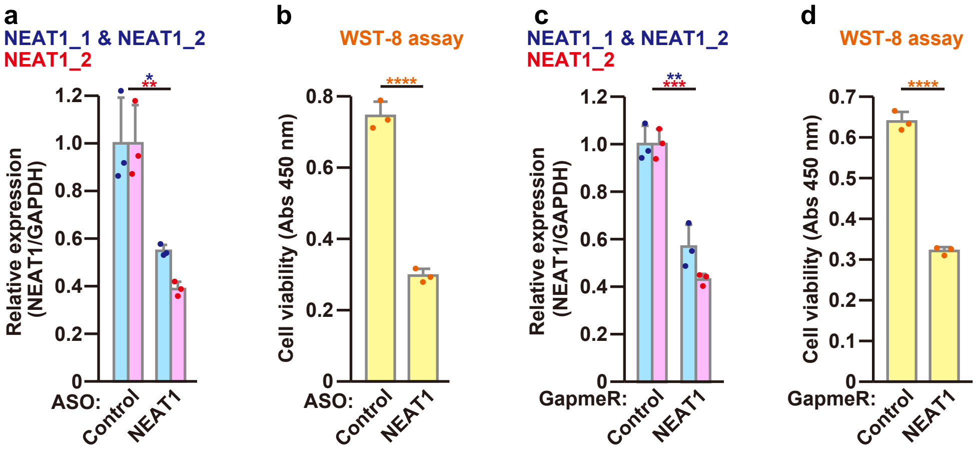

a-d NSC-34 cells were transfected with 30 nM control or NEAT1 antisense oligonucleotide (ASO) (a, b) or with 30 nM antisense LNA GapmeR control or NEAT1 (GapmeR) (c, d). At 60 h after the transfection, the quantitative real-time PCR analysis of NEAT1 was performed (a, c). The cell viability was detected by WST-8 assay (b, d). The data are presented as means ± SD (N = 3). Statistical analysis was performed by the unpaired t test.

Fig. S7 HnRNPM does not activate NEAT1 promoter or stabilize exogenously expressed NEAT1_1.

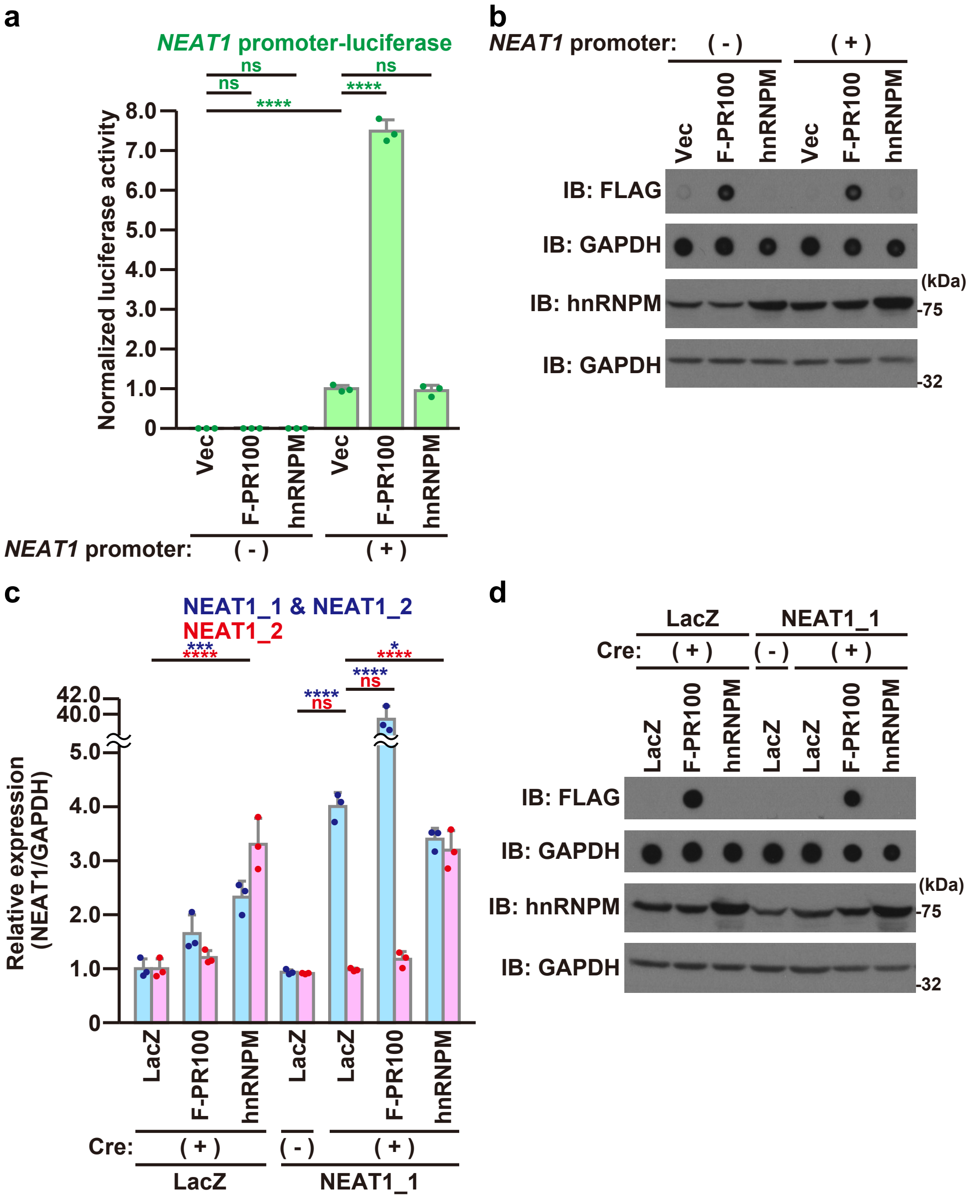

a, b NSC-34 cells were transfected with the *NEAT1*-promoter (+) or *NEAT1*-promoterless (-) luciferase vector together with the pEF1-Myc/His-vec (Vec), the pEF1-FLAG-PR100 (F-PR100), or -hnRNPM. At 48 h after the transfection, the luciferase activity was measured (a). The cell lysates were subjected to immunoblotting (IB) and dot blotting analysis using indicated antibodies (b). The data are presented as means ± SD (N = 3). Statistical analysis was performed by one-way ANOVA followed by the Tukey's multi comparisons test.

c, d NSC-34 cells were infected with adenovirus encoding LacZ or mouse NEAT1_1 at an MOI of 10. Cells were also co-infected with adenovirus encoding FLAG-PR100 (F-PR100) at an MOI of 200 or hnRNPM at an MOI of 400 together with adenovirus encoding LacZ (-) or Cre-recombinase (+) at an MOI of 40. To keep the constant total MOIs of adenoviruses, appropriate MOIs of LacZ-encoding adenovirus were added for each infection. At 48 h after the infection, the quantitative real-time PCR analysis of NEAT1 was performed (c). The cell lysates were subjected to immunoblotting (IB) and dot blotting analysis using indicated antibodies (d). The data are presented as means ± SD (N = 3). Statistical analysis was performed by one-way ANOVA followed by the Dunnett's multi comparisons test. For appropriate comparison, the NEAT1_1/Cre/F-PR100 samples were omitted for statistical analysis of NEAT1_1 due to the existence of one sample with extremely large value in the NEAT1_1/Cre/F-PR100 samples. The comparison between NEAT1_1/Cre/F-PR100 sample and one of the other NEAT1_1 samples was performed by one-way ANOVA followed by the Dunnett's multi comparisons test. In this condition, poly-PR did not increase the expression of hnRNPM probably due to the less expression of poly-PR achieved by the infection with the low MOI.

Fig. S8 Poly-PR suppresses the function of hnRNPF and the overexpression of either hnRNPF or hnRNPH1 causes neurotoxicity.

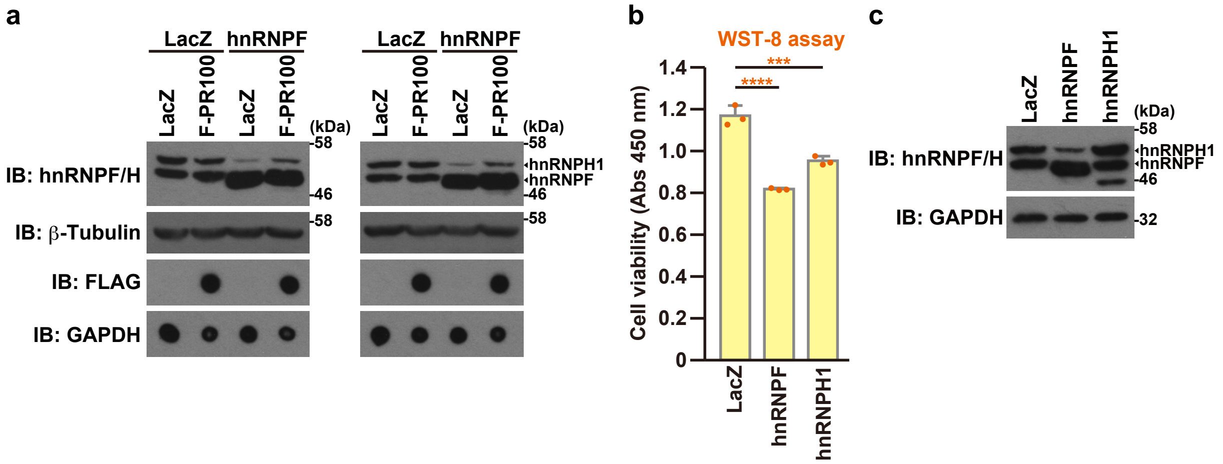

a NSC-34 cells were infected with adenovirus encoding LacZ or hnRNPF at an MOI of 200. Cells were also co-infected with adenovirus encoding LacZ or FLAG-PR100 at an MOI of 400. At 48 h after the infection, the cell lysates were subjected to immunoblotting (IB) and dot blotting analysis using indicated antibodies.

b, c NSC-34 cells were infected with adenovirus encoding LacZ, hnRNPF, or hnRNPH1 at an MOI of 400. At 48 h after the infection, the cell viability was detected by WST-8 assay (b). The cell lysates were subjected to immunoblotting (IB) using indicated antibodies (c). The data are presented as means ± SD (N = 3). Statistical analysis was performed by one-way ANOVA followed by the Dunnett's multi comparisons test.

Fig. S9 Poly-PR binds to TDP-43 and RBM14 through the low-complexity domain (LCD).

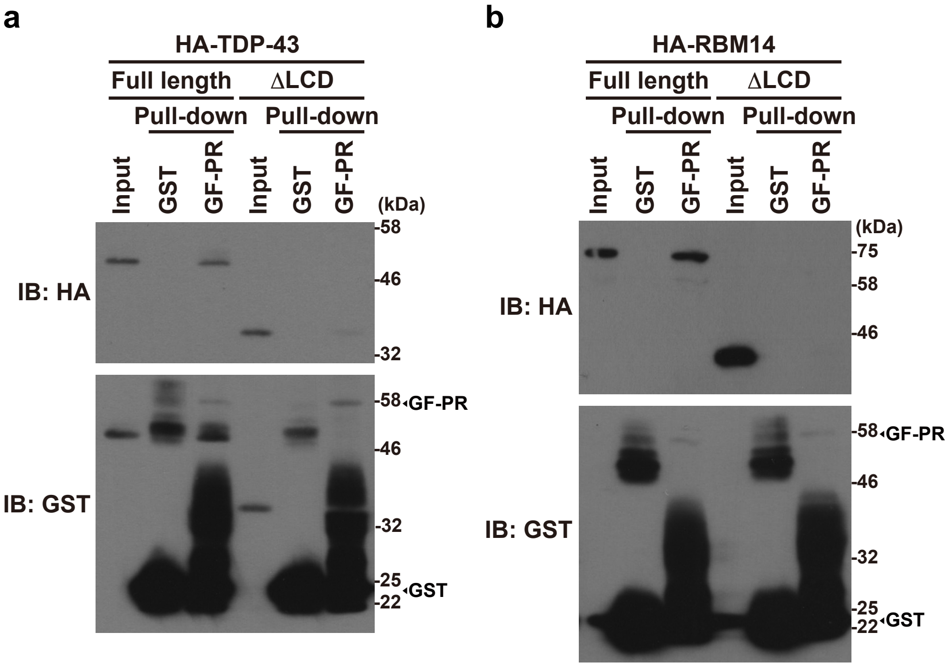

a, b Lysates of NSC-34 cells overexpressing HA-tagged TDP-43-wt (Full length), TDP-43-(1-273) (ΔLCD) (a), RBM14-wt (Full length), or RBM14-(1-349) (ΔLCD) (b) were mixed with purified recombinant GST or GST-FLAG-PR100 (GF-PR)-bound glutathione beads. After rotation at 4 ºC, the glutathione beads were washed and were subjected to immunoblotting (IB) using indicated antibodies. In western blotting using anti-GST antibody, the large smear within the molecular weights ranging 25–46 kDa in the GF-PR lane is thought to consist of C-terminal truncated GST-FLAG-PR100 proteins. A band located around 50 kDa in the GST lane is thought to represent dimerized and/or aggregated GST-derived proteins.

Table S1 A summary of paraspeckle proteins identified as poly-PR-binding protein

Band No.	Protein name	Accession	Total score	Sequence coverage (%)	No. of peptides matched
5	Matrin-3	Q8K310	12.9	11.9	7
7	Splicing factor, proline- and glutamine-rich	Q8VIJ6	20.1	20.7	12
10	Heterogeneous nuclear ribonucleoprotein M	Q9D0E1	20.5	19.5	14
	RNA-binding protein 14	Q8C2Q3	12.3	11.8	7
11	Protein FAM98A	Q3TJZ6	22.5	30.3	13
	Non-POU domain-containing octamer-binding protein	Q99K48	14.4	22.0	8
12	Heterogeneous nuclear ribonucleoprotein H	O35737	25.4	39.0	17
13	Heterogeneous nuclear ribonucleoprotein F	Q9Z2X1	13.6	26.8	9

Band numbers correspond to those in Suzuki *et al.*, Cell Death & Disease (2018) 9:975, Figure 6a. A "total score" is a value for the level of confidence in the protein identification, calculated by ProteinPilot. It can be converted to a percentage confidence score using the following formula: ProtScore = -log(1-percentage confidence/100). As an approximate guide, ProteinPilot total scores give the following percentage levels of confidence; score >2 (>99% confidence), score >3 (>99.9% confidence). A "sequence coverage (%)" is a percentage of an identified protein that is covered by all matched peptides.
